# Electron localization in a mixed-valence diniobium benzene complex†
†Electronic supplementary information (ESI) available. CCDC 1022634–1022637. For ESI and crystallographic data in CIF or other electronic format see DOI: 10.1039/c4sc02705a


**DOI:** 10.1039/c4sc02705a

**Published:** 2014-11-11

**Authors:** Thomas L. Gianetti, Grégory Nocton, Stefan G. Minasian, Nikolas Kaltsoyannis, A. L. David Kilcoyne, Stosh A. Kozimor, David K. Shuh, Tolek Tyliszczak, Robert G. Bergman, John Arnold

**Affiliations:** a Department of Chemistry , University of California , Berkeley , CA 94720 , USA . Email: arnold@berkeley.edu ; Email: rgbergman@berkeley.edu; b Laboratoire de Chimie Moléculaire , CNRS , Ecole Polytechnique , 91128 Palaiseau , France . Email: greg.nocton@polytechnique.edu; c Chemical Sciences Division , Lawrence Berkeley National Laboratory , Berkeley , CA 94720 , USA; d Chemistry Division , Los Alamos National Laboratory , Los Alamos , NM 87545 , USA; e Department of Chemistry , University College London , 20 Gordon Street , London , WC1H0AJ , UK . Email: n.kaltsoyannis@ucl.ac.uk; f Advanced Light Source , Lawrence Berkeley National Laboratory , Berkeley , CA 94720 , USA

## Abstract

Reaction of the neutral diniobium benzene complex {[Nb(BDI)N^*t*^Bu]_2_(μ-C_6_H_6_)} (BDI = *N*,*N*′-diisopropylbenzene-β-diketiminate) with Ag[B(C_6_F_5_)_4_] results in a single electron oxidation to produce a cationic diniobium arene complex, {[Nb(BDI)N^*t*^Bu]_2_(μ-C_6_H_6_)}{B(C_6_F_5_)_4_}. Investigation of the solid state and solution phase structure using single-crystal X-ray diffraction, cyclic voltammetry, magnetic susceptibility, and multinuclear NMR spectroscopy indicates that the oxidation results in an asymmetric molecule with two chemically inequivalent Nb atoms. Further characterization using density functional theory (DFT) calculations, UV-visible, Nb L_3,2_-edge X-ray absorption near-edge structure (XANES), and EPR spectroscopies supports assignment of a diniobium complex, in which one Nb atom carries a single unpaired electron that is not largely delocalized on the second Nb atom. During the oxidative transformation, one electron is removed from the δ-bonding HOMO, which causes a destabilization of the molecule and formation of an asymmetric product. Subsequent reactivity studies indicate that the oxidized product allows access to metal-based chemistry with substrates that did not exhibit reactivity with the starting neutral complex.

## Abstract 2

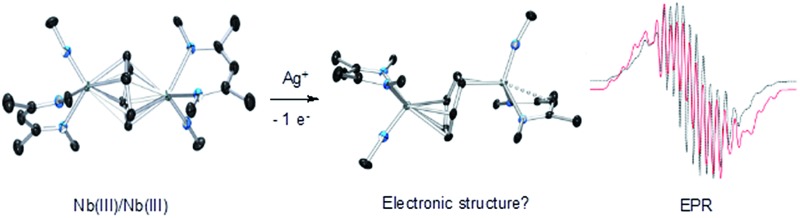
One electron oxidation of a neutral diniobium benzene complex leads to a mixed-valence species. Single crystal X-ray diffraction, EPR, L_3,2_-edge XANES, and DFT indicate that the unpaired electron is localized on one metal center.

## Introduction

Since the discovery of metallocenes, the bonding modes and structures of organometallic molecules have been an important facet of chemistry over the last sixty years.^1–4^ Among these molecules, arene complexes have drawn particular attention. Species that contain an arene coordinated in between two metal ions – bimetallic arene complexes – have been highly sought since the first report of a divanadium benzene complex [CpV]_2_(μ-η^6^:η^6^-C_6_H_6_) thirty years ago.^5^ Not only are they fascinating molecules from a structural perspective, they also provide a starting platform for further reactivity and open new synthetic routes for the synthesis of highly reactive complexes.^6^ To-date, bimetallic arene complexes containing many different metal ions from the s-, p-, d- and f- block elements have been prepared and studied, and potential applications in the development of organic transformations and materials synthesis have been demonstrated.^4,7^

Bonding in bimetallic arene complexes has been described by moderate π-donation from the arene to the metal, with some δ-type M → arene donation from the metal d- or f-orbitals to unoccupied orbitals of the arene.^8–10^ However, cleavage occurs at the metal center in reactions with chemical oxidants, suggesting that the arene ring acts as an electron reservoir in support of the reactive and highly electron rich metal centers.^6,9^ Recently, P. Arnold *et al.* provided the first example of direct arene functionalization using a neutral diuranium arene complex with the report of a remarkable direct C–H borylation.^11^ A key point in this reaction is a formal disproportionation that allows the arene to be reduced. The idea of a formal disproportionation, in which a single electron is transferred to another metal in a bimetallic process, is of great importance in bimetallic arene complexes because their high symmetry suggests that they always provide two electrons to react, one from each metal center, so that radical chemistry or single electron transfer (SET) is not expected. A strategy for accessing radical chemistry with such complexes is to synthesize electronically unsymmetrical bimetallic arene complexes, *i.e.* mixed-valence arene complexes, in which an unpaired electron may be either localized on one metal center or delocalized across both.^12^ The initial report in 1969 by Creutz and Taube of the mixed-valence quintuply charged decaammine (μ-pyrazine) bisruthenium ion^13^ has inspired hundreds of publications,^14,15^ and a number of debates over the electronic structure of these types of molecules.^16–21^ To the best of our knowledge, three mixed-valence arene complexes, two of uranium and one of titanium, have been reported,^9,22,23^ however the extent of delocalization and changes in chemical reactivity have not been fully addressed.^9,22,23^ Notably, Mazzanti and coworkers recently showed that reactivity between K^+^ and a neutral diuranium benzene complex causes disproportionation and formation of a mixed valence benzene complex.^22^ In the present report, the reason for the stability of such mixed valence species, whether it comes from the nature of the bonding with the arene ring (delocalization) or from a counter-cation that provides structural and/or electronic stability, is addressed.

**Scheme 1:**
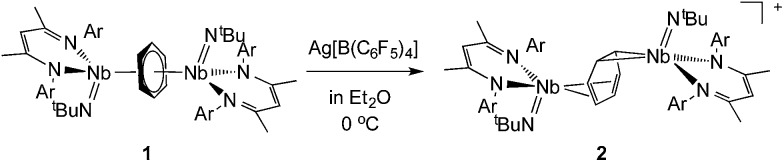


In previous work we described the synthesis and kinetics for the formation of a diniobium benzene complex {[Nb(BDI)N^*t*^Bu]_2_(μ-C_6_H_6_)}, **1** (BDI = *N*,*N*′-diisopropylbenzene-β-diketiminate, Scheme 1), that was found to be unreactive with most small molecules.^24^ This surprising observation led us to probe its redox behavior as a potential means to activate the molecule toward further reactivity. Here, we present the one-electron oxidation of **1** that leads to the formation of a stable cationic mixed-valence diniobium arene complex {[Nb(BDI)N^*t*^Bu]_2_(μ-C_6_H_6_)}{B(C_6_F_5_)_4_}, **2**. Techniques including X-ray crystallography, NMR spectroscopy, and cyclic voltammetry (CV) have been used to elucidate the solid state and solution structures, while DFT calculations with GGA and hybrid functionals, together with solid-state magnetometry, EPR, UV-vis and X-ray absorption spectroscopies have probed electron localization in the complex. Combined, the in-depth study provides the insight necessary to understand the nature of enhanced reactivity for the two chemically inequivalent Nb atoms.

## Results and discussion

### Synthesis and X-ray analysis

The oxidation of the arene complex **1** with Ag[B(C_6_F_5_)_4_] in Et_2_O led to a color change from deep red to deep green within 10 minutes, followed by precipitation of a yellow/green microcrystalline powder within an hour. The solid residue obtained after removal of the reaction solvent was washed several times with Et_2_O and extracted with α,α,α-trifluorotoluene. Crystallization at –35 °C afforded green crystals that were identified as the one-electron oxidation product {[Nb(BDI)N^*t*^Bu]_2_(μ-C_6_H_6_)}{B(C_6_F_5_)_4_}, **2** (Scheme 1), by single crystal X-ray diffraction, electrochemistry, magnetic studies and NMR spectroscopy.

**Fig. 1:**
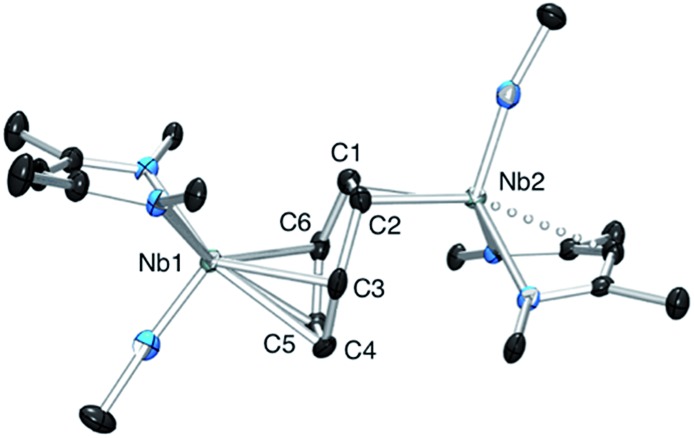
Thermal ellipsoid plot of the cationic part of **2**, {[Nb(BDI)N^*t*^Bu]_2_(μ-C_6_H_6_)}^+^. ^*i*^Pr_2_Ar groups of BDI ligand, CH_3_ groups of ^*t*^BuN ligand, counter ion [B(C_6_F_5_)_4_]^–^ and hydrogens have been removed for clarity. Thermal ellipsoids are represented at 50%. Selected bond distances in Å: Nb(2)–C(1), 2.225(5); Nb(2)–C(2), 2.226(5); Nb(1)–C(3), 2.466(5); Nb(1)–C(6), 2.384(5); Nb(1)–C(4), 2.397(5); Nb(1)–C(5), 2.480(5); C(1)–C(2), 1.520(7); C(2)–C(3), 1.460(7); C(3)–C(4), 1.390(8); C(4)–C(5), 1.446(8); C(5)–C(6), 1.382(8); C(6)–C(1), 1.454(7).

X-ray quality crystals of complex **2** were obtained from recrystallization in DCE, 1,2-dichloroethane, at –15 °C overnight (Fig. 1). Unlike **1**,^24^ in which the bridged benzene is symmetrically bound to the two metal centers *via* a μ^2^-η^6^:η^6^ coordination mode, **2** is best described as having a μ-η^2^:η^4^ coordination mode, which is unprecedented to the best of our knowledge. This leads to a dramatic increase in the Nb–Nb separation to 4.656(4) Å in **2** as compared to 3.914(5) Å in **1**. The benzene ring is slightly distorted with a mean deviation from planarity of 0.07 Å, a dihedral angle of 14.9° and an alternation of C–C bond distances. While C(2)–C(3), (1.460(7) Å), C(3)–C(4), (1.390(8) Å), C(4)–C(5), (1.446(8) Å), C(5)–C(6), (1.382(8) Å) and C(6)–C(1) (1.454(7) Å) follow typical alternation of single and double bonds, C(1)–C(2) is significantly elongated (1.520(7) Å), so that Nb(1) appears to interact with a butadiene-like fragment of the arene, and Nb(2) is coordinated to the arene *via* C(1) and C(2). This picture is reinforced by the short Nb(2)–C_av_ bond length of the η^2^-interaction (2.22 Å) compared to the longer Nb(1)–C_av_ distance to the four C atoms engaged in the η^4^-binding (2.43 Å).

The Nb(1)–N(BDI) distances of 2.152(4) Å and 2.144(4) Å differ slightly from the Nb(2)–N(BDI) distances of 2.177(4) Å and 2.191(4) Å in agreement with the altered coordination environment. Additionally, the distortion of the BDI moiety around Nb(2) and the Nb(2)–C(31) (BDI) distance of 2.638(9) Å suggests the presence of an intramolecular donation from the π-system of the BDI ligand into the metal center (Fig. 1, dashed line), supporting a strong metal–arene interaction between the d electrons of Nb(2) atom and the empty π* orbital of the C(1)–C(2) ethylene-like fragment. This intramolecular interaction between the BDI backbone and the metal center has been observed previously in Zr(iv),^25–27^ Ti(iv),^28^ and lanthanide complexes.^29^ As was observed in **1**, the Nb=N(^*t*^Bu) groups are *trans* to each other to minimize steric interactions.

### Electrochemistry

**Fig. 2:**
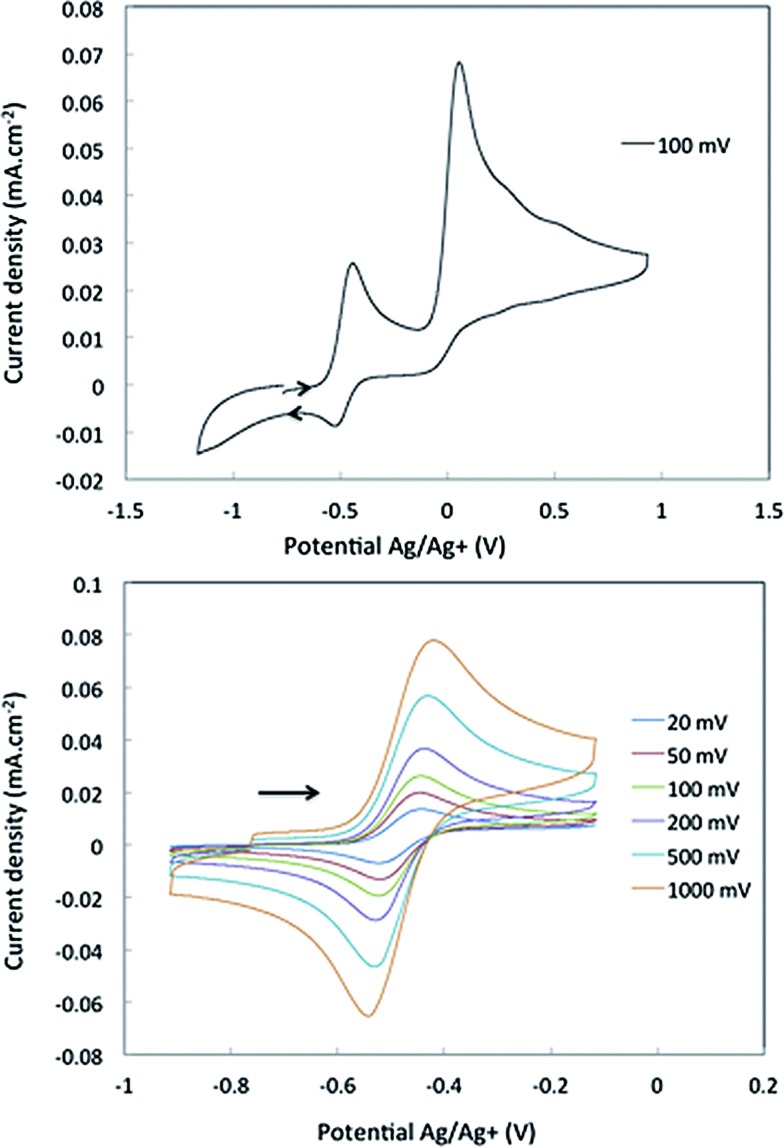
Cyclic voltammetry of complex **1** in α,α,α-trifluorotoluene: top, oxidation of **1** over a large range of potential; bottom, one electron reversible wave of **1** at different scan-rates.

Cyclic voltammetry studies were carried out on complexes **1** and **2** in α,α,α-trifluorotoluene. For **1**, two waves are observed (Fig. 2). The first is a reversible process with *E*_1/2_ = –0.48 V *vs.* Ag/Ag^+^ (–0.93 V *vs.* Fc^+^/Fc, Fig. 2 right and S7†) while the second is irreversible with an oxidation potential of *E*_a_ = +0.04 V *vs.* Ag/Ag^+^ (–0.41 V *vs.* Fc^+^/Fc). Integration of the current wave compared to an equimolar solution of **1** and Cp_2_Co is indicative of a one-electron oxidation (Fig. S8†), while the second wave is an irreversible two-electron oxidation. Upon reduction, the cyclic voltammogram of **2** shows the same reversible peak at *E*_1/2_ = –0.48 V *vs.* Ag/Ag^+^, allowing the assignment of this signal to the one-electron process that converts **1** to **2** (Fig. S10†). This half-potential is within the range reported for the reversible electrochemical oxidation of a series of Nb^3+^ complexes (η^5^-C_5_H_4_SiMe_3_)_2_NbCl(L) (L = phosphine or alkyne, *E*_1/2_ = [–1.69 to –0.89] V *vs.* Fc^+^/Fc),^30^ and is consistent with a metal-based one electron oxidation.

### Magnetic study and NMR spectroscopy

Magnetic data for **2** were measured in the solid state using a SQUID magnetometer over the temperature range 5–300 K (see Fig. S15 and S16†). The effective moment at room temperature is 1.8 μ_eff_, in agreement with *S* = 1/2 and one unpaired electron and is persistent over the entire temperature regime. As expected for a paramagnetic species, the ^1^H NMR spectrum of **2** exhibits broad peaks that shift with changes in temperature (253 K to 373 K, see Fig. S13 and S14†), along with the very fast longitudinal relaxation times (*T*_1_ = [5–12.7] ms, Fig. S12†).^31,32^ However, the ^1^H NMR spectrum is unusual in that all the resonances are located close to the typical diamagnetic region (12 to –5 ppm) and the small number of peaks (8) along with their relative integrations are not consistent with the asymmetry observed in the solid state. Additionally, no signals are observed in the ^13^C NMR spectrum after 12 h of acquisition.

To understand this NMR spectrum, an experiment was conducted wherein an equimolar amount of **2** and trimethoxybenzene (a non-reacting internal standard) were combined and analysed by ^1^H NMR spectroscopy for the purposes of comparing their relative peak intensities. This experiment revealed that only half of the number of hydrogens expected for **2** are observed in the ^1^H NMR spectrum, corresponding to a single BDI ligand and one ^*t*^Buimido group. The other BDI ligand and ^*t*^Buimido ligand as well as the μ-benzene are not observed. This has been further confirmed by NMR spectroscopic analyses of the deuterated benzene analog **2-d_6_**, which exhibits a similar spectrum to **2** by ^1^H NMR spectroscopy, but no resonance corresponding to C_6_D_6_ is detected in the ^2^H NMR spectrum.

This behavior suggests that the single electron is mostly localized on one niobium atom on the NMR time scale. In this model, the protons that are the closest to the paramagnetic center relax very quickly and their chemical shifts are strongly shifted mostly because of the pseudo-contact term,^32^ which results in complete broadening of their signals. On the other hand, the nuclei that lie the farthest from the paramagnetic metal center are less influenced by the pseudo-contact term and are therefore more likely observed in the typical diamagnetic region. This NMR spectroscopic analysis further confirms that the two niobium metal centers of **2** are electronically inequivalent, but the exact extent of the delocalization and the impact of Fermi contact term is difficult to ascertain with any degree of precision.

On the basis of the solid-state structure and solution-phase electrochemistry, magnetism, and NMR spectroscopy data, the reaction of complex **1** with Ag[B(C_6_F_5_)_4_] results in a formal one electron oxidation to form a new asymmetric complex **2** in which one of the Nb atoms carries an unpaired electron. To probe the reactivity of **2** in a rational manner, we first sought additional insights regarding the exact nature of electron localization from DFT calculations and further spectroscopic studies.

### DFT calculations

DFT calculations employing the PBE (GGA) functional were conducted to provide a framework for evaluating the electronic structure of the diniobium arene complexes **1** and **2**. The geometries of both complexes were optimized from their X-ray structures; a spin restricted closed shell electronic structure was assumed for **1** and an unrestricted open shell doublet for **2**. The correlation between calculated and experimental bond lengths is reasonable (see metrical parameters in the ESI†). As both complexes feature distorted benzene ligands, the energy differences between free benzene and the benzene fragments in **1** and **2** were calculated; in the complexes the benzene rings are 23.7 kcal mol^–1^ and 22.7 kcal mol^–1^ less stable, respectively, than in free benzene. Moreover, the average C–C distance in the benzene ring in **1** is 1.458 Å as compared to 1.394 Å in free benzene. Taken together, these observations suggest strong Nb → benzene charge transfer in **1**, which is supported by the partial atomic charge data in Table 1. Although the absolute values of the charges differ significantly between the different approaches, all three indicate substantial build up of electron density on the arene ligand.

**Table 1:** Partial atomic charges on the niobium atoms and benzene fragments in **1** and **2**, calculated using the Mulliken, Hirshfeld and quantum theory of atoms in molecules (QTAIM) approaches

		Mulliken	Hirshfeld	QTAIM
**1**	Nb(1)	2.89	0.63	1.88
	Nb(2)	2.90	0.63	1.87
	Benzene	–2.46	–0.38	–1.16
**2**	Nb(1)	2.82	0.68	1.87
	Nb(2)	2.68	0.68	1.92
	Benzene	–1.69	–0.25	–0.79

**Fig. 3:**
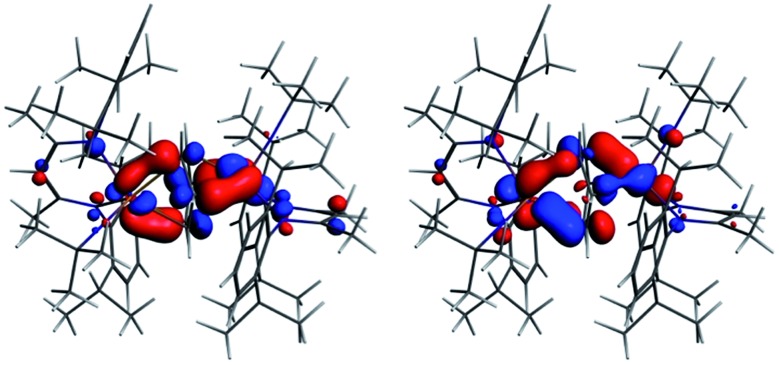
HOMO-1 [–3.934 eV: 13.0% Nb(1) d, 17.1% Nb(2) d, 1.1% Nb(2) s, 38.1% C (benzene) p] and HOMO [–3.875 eV: 20.8% Nb(1) d, 17.1% Nb(2) d, 35.0% C (benzene) p] of **1**. Isosurface value = 0.05.

The interaction energy between the two Nb fragments and the benzene ring in **1** has been calculated using the Ziegler–Rauk energy decomposition scheme.^33^ The large total bonding energy (142.9 kcal mol^–1^) and the fact that the orbital interaction term contributes 47% of the overall stabilizing contribution, reinforce the conclusion of a strong interaction between the arene ring and the metal centre. Consistent with such an interaction, and that it features metal → benzene charge transfer, are the canonical HOMO-1 and HOMO shown in Fig. 3. Both orbitals are clearly δ-backbonding and show that oxidation to form **2** would disrupt the Nb–arene interaction.

In **2**, the C_6_ axis that defines the center of the benzene ring is not aligned with the Nb–Nb axis as it is the case for **1**. Interestingly, when the geometry optimization of **2** was performed starting from the optimized structure of **1**, removing one electron and employing the spin unrestricted approach, the calculation converged to a different geometry, optimized structure **2-II**, than that found when starting from the solid-state structure of **2**, optimized structure **2-I**. This symmetrical optimized geometry, **2-II**, for the cationic arene complex is only 2.4 kcal mol^–1^ less stable than the previously optimized **2-I**.

**Fig. 4:**
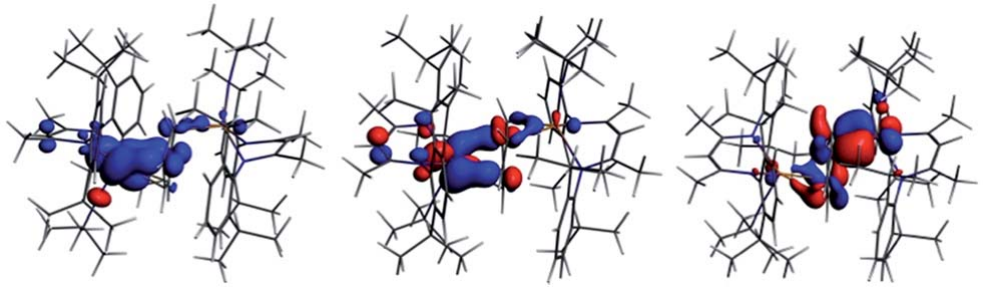
Spin density of **2-I**. [left, blue = α, red = β, isosurface = 0.003], SOMO [middle, –6.349 eV. 41.4% Nb(1) d, 1.6% Nb(1) s, 2.5% Nb(2) d, 1.4% Nb(2) p, 23.0% C (benzene) p] and α HOMO [right, –6.941 eV: 3.9% Nb(1) d, 26.2% Nb(2) d, 1.4% Nb(2) p, 42.4% C (benzene) p].

Fig. 4 shows the spin density, the SOMO and α HOMO of **2**. The spin density indicates much greater localization of the unpaired electron on Nb(1) (0.66) than Nb(2) (0.06), with significant spin density located on carbons of the benzene ring (0.14 for C3 and 0.15 for C6, respectively). The SOMO is much more localized on Nb(1) than on Nb(2), as is the spin density, such that both niobium ions contribute, but not equally. Both the α and β components of the HOMO show a very strong interaction between two carbon atoms of the benzene and Nb(2) so that the C–Nb(2)–C unit may be described as a metallacyclopropane. This explains the longer distance found for C(1)–C(2) of 1.520(7) Å compared to the other C–C bonds of the arene moiety. Returning to Table 1, all three charge schemes indicate that the largest change between **1** and **2** is for the benzene ring, suggesting that some of the charge build up on the ring arising from δ backbonding in the neutral complex is pulled back in the cation. The Mulliken data suggest that this leads to a reduction in the positive charge of the metals from **1** to **2**, while the other two methods indicate little change in the metal charge.

In summary, the DFT calculations suggest a highly covalent interaction between the Nb atoms and benzene ligand in **1**, arising primarily from Nb → arene δ back bonding. This covalent interaction leads to substantial charge build up on the benzene ligand with a concomitant structural distortion of the ring. Oxidation to **2** leads to an asymmetric geometric and electronic structure with the unpaired electron localized primarily on Nb(1).

### XANES studies

Of the experimental approaches to probe electronic structure in d-block metal complexes, L_3,2_-edge XANES (X-ray absorption near-edge structure) spectroscopy is particularly effective because it probes the valence d-orbitals directly with dipole allowed-transitions from core metal 2p-orbitals. Niobium L_3,2_-edge absorptions arise from dipole allowed 2p^6^4d^*n*^ → 2p^5^4d^*n*+1^ transitions that are split by roughly 94 eV into L_3_ (2p_3,2_ → 4d_5/2_ and 2p_3,2_ → 4d_3/2_) and L_2_ (2p_1/2_ → 4d_3/2_) edges resulting from spin-orbit coupling of the core hole.^34^ The integrated intensity of the L_3,2_-edge transitions can be related to the occupancy of the d-orbitals by dividing by the continuum intensity using established methodology.^35,36^ In this study, the occupancy of the Nb 4d-orbitals in **2** was determined by measuring the Nb L_3,2_-edge XANES spectra in transmission using a scanning transmission X-ray microscope (STXM).

**Fig. 5:**
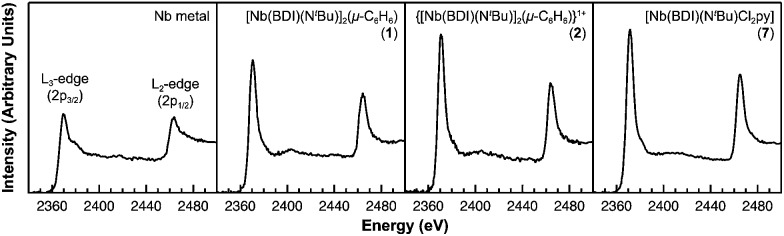
Representative background subtracted Nb L_3,2_-edge XANES spectra collected in transmission using STXM for Nb metal and compounds **1**, **2**, and **7**. Spectra for compounds **1**, **7**, and Nb metal are adapted with permission from T. L. Gianetti, G. Nocton, S. G. Minasian, N. C. Tomson, A. L. D. Kilcoyne, S. A. Kozimor, D. K. Shuh, T. Tyliszczak, R. G. Bergman and J. Arnold, *J. Am. Chem. Soc*., 2013, **135**, 3224–3236. Copyright 2013 American Chemical Society.

**Fig. 6:**
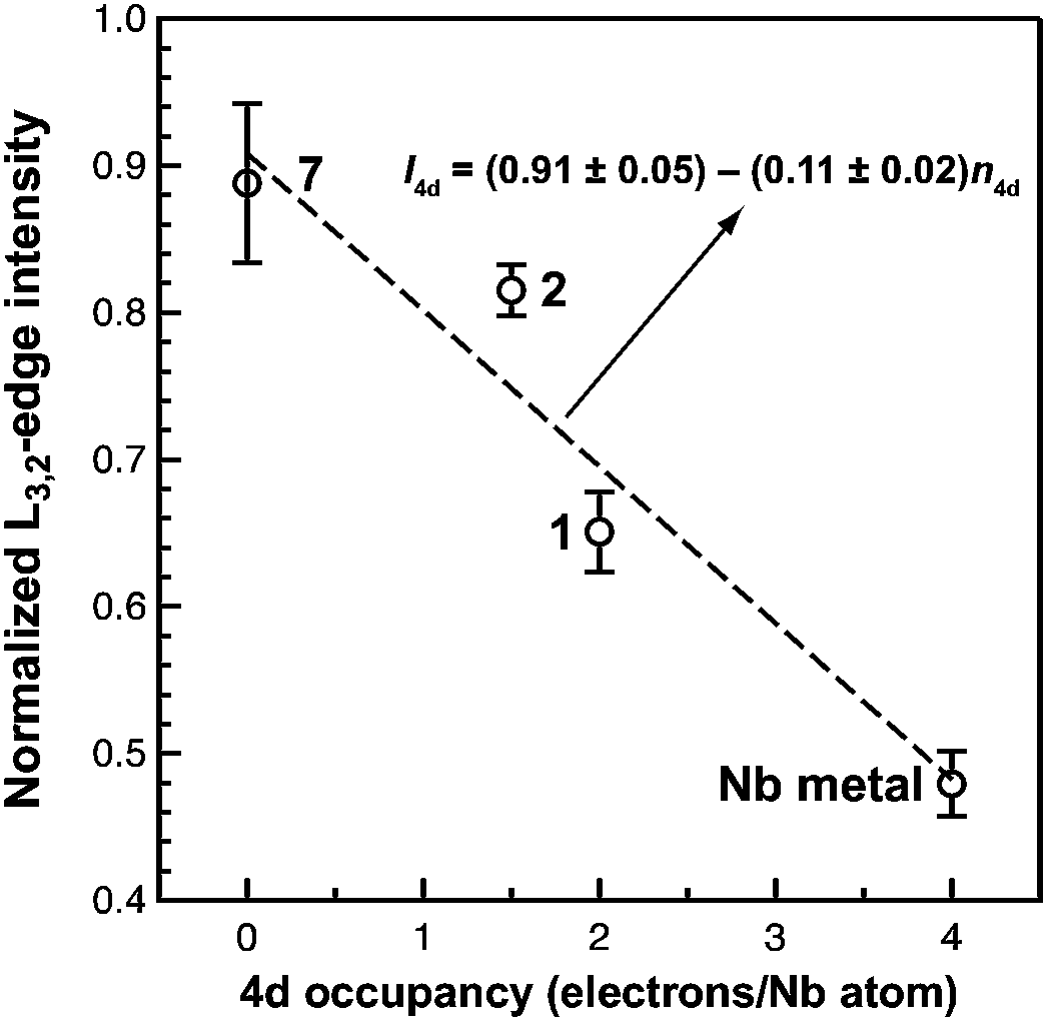
A plot of normalized L_3,2_-edge intensity *vs.* 4d occupancy of {[Nb(BDI)N^*t*^Bu]_2_(μ-C_6_H_6_)} (**1**), {[Nb(BDI)N^*t*^Bu]_2_(μ-C_6_H_6_)}{B(C_6_F_5_)_4_} (**2**), [Nb(BDI)(N^*t*^Bu)Cl_2_py] (**7**), and Nb metal. Error bars are taken from the standard deviation in the intensities determined over repeated measurements. A linear fit to the data (dashed line) gives the equation shown.

Transmission measurements made using STXM provide accurate peak intensities in a non destructive manner and are compatible with air-sensitive inorganic materials.^37–40^ In previous work, this method was employed to assign (4d^2^)(4d^2^) electronic configurations to both Nb atoms in **1**, which required a formally neutral bridging arene ligand.^24^ The background-subtracted Nb L_3,2_-edge spectra for **2** and reference materials Nb metal, neutral **1**, and [Nb(BDI)N^*t*^BuCl_2_py], **7** (ref. 24) are given in Fig. 5. Although the solid-state X-ray crystal structure of **2** shows two distinct Nb chemical environments, distinct peaks are not resolved in the L_3,2_-edge spectrum of **2**, as a possible consequence of the energy resolution (full width at half maximum, fwhm) at the Nb L_3,2_-edge, which was estimated at 1.0 eV.^41^ For the reference materials, the normalized intensity of the L-edge transitions increases as the d-orbital occupancies decrease from Nb metal (4d^4^5s^1^, 0.48(2)), to fully-oxidized **7** (4d^0^, 0.89(5)). A decrease in the 4d-orbital occupancy from neutral **1** to cationic **2** is reflected by an increase in the edge intensity from 0.65(3) to 0.82(2), respectively. These results suggest that formation of complex **2** is best described by a single-electron oxidation of complex **1** (4d^2^)(4d^2^) to produce a complex with a (4d^2^)(4d^1^) electronic configuration (mixed +3 and +4 formal oxidation states). Using this assignment, plots of the L_3_-edge adsorption energies and normalized intensities relative to 4d-orbital occupancies were developed (Fig. 6).

Linear fits to the data show that a (4d^2^)(4d^1^) electronic configuration is most likely within the oxidation state formalism, however, invoking a non-integral increase in Nb charge in **2** resulting from Nb → arene δ-donation or bonding with the imido ligand improves the correlation.

### UV-vis spectroscopy

Electronic spectroscopy is often used to evaluate the extent of electron delocalization in mixed valence Creutz–Taube ions by focusing on the presence (or absence), and intensity of the intervalence charge transfer band (IVCT).^16^

Three classes are used to describe the extent of electron delocalization over the two different metal centres. Class I complexes present no IVCT bands and no delocalization occurs on the time scale of the electronic transition. Conversely, delocalization is important in class III compounds as shown by a sharp and intense band in the visible or near infrared region. The IVCT bands are also present for class II compounds, but they are broader and less intense which suggests that the unpaired electron is in an intermediate state between fully localized and delocalized.

**Fig. 7:**
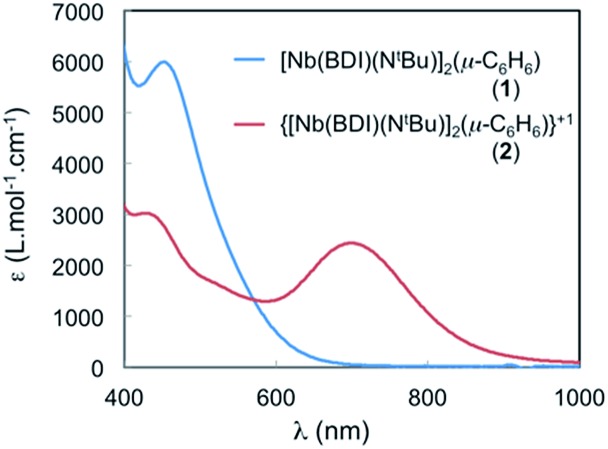
Visible spectra of **1** and **2** in α,α,α-trifluorotoluene at room temperature. Electronic absorbance spectrum of 47.8 μM solution of **1** and 38.2 μM solution of **2** in α,α,α-trifluorotoluene. Complex **2**: 326 nm (27 000 cm^–1^ M^–1^); 426 nm (3200 cm^–1^ M^–1^); 704 nm (2430 cm^–1^ M^–1^).

The spectrum of **2** in toluene features a broad, Gaussian shaped and relatively strong band (*ε* ∼ 2500 cm^–1^ M^–1^) centred at 700 nm (14 285 cm^–1^) and another at 430 nm (23 250 cm^–1^) (see Fig. 7). The large absorbance of these features is inconsistent with Laporte forbidden d–d transitions and could involve an IVCT processes. To gain further insight into the nature of these transitions, TD-DFT calculations were performed employing the B3LYP functional on **2** using the calculated optimized geometry, **2-I**, that fits the X-ray crystal structure data and that was described in the DFT section (see Fig. S18†). Two transitions are predicted at around 450 nm and are in agreement with MLCT transitions involving the benzene ligand. The presence of a similar transition in the neutral and symmetric complex **1** is in agreement with this assignment (Fig. 7, blue curve). In addition, another transition is observed at 653 nm in the TD-DFT calculations and is blue shifted compared to that at 700 nm, observed in the experiment, but still in good agreement with the calculation (653 nm *vs.* 700 nm). The TD-DFT suggests that this transition near 700 nm involves electron transfer from the SOMO (localized on Nb(1), see above) to a vacant orbital with predominantly Nb(2) character. Hence the transition at 700 nm is assigned to a formal d–d transition in which the single electron is transferred between the two Nb atoms *via* an IVCT. The band is relatively broad, with *H*_ab_ values of 1500 cm^–1^ and *α* ^2^ of 0.011, which indicates that **2** is a class II mixed valence system.^42^

### EPR spectroscopy

**Fig. 8:**
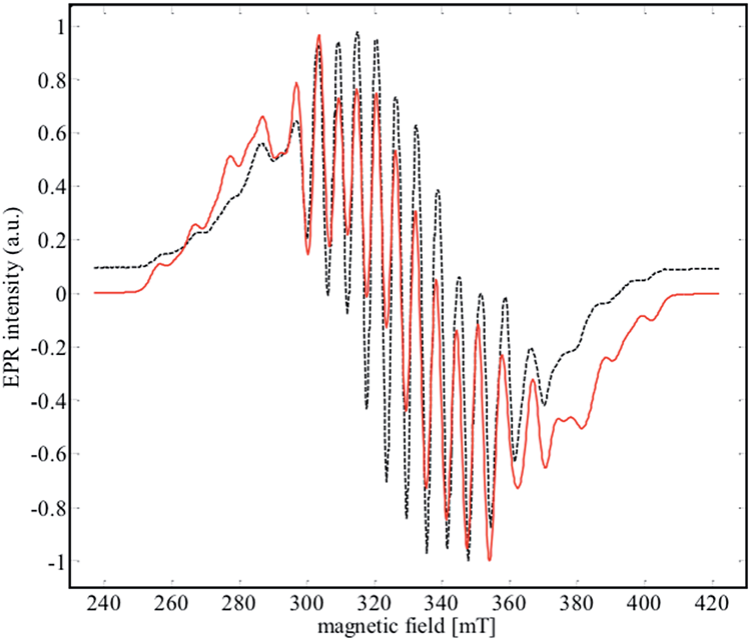
X-Band EPR spectrum (black dashed line) of **2** recorded in frozen solution of α,α,α-trifluorotoluene at 4 K. The red line corresponds to the simulated data for the parameters given in Table 2.

EPR spectroscopy was used to experimentally determine the location of the unpaired electron and evaluate the extent of delocalization. An X-band EPR spectrum of **2** was recorded in the solid state at both 298 K and 4 K (see Fig. S17†), and in a frozen solution (4 K) of α,α,α-trifluorotoluene (Fig. 8).

Niobium has an *I* = 9/2 nuclear spin, so a 10-line pattern is expected if the spin is located on a single metal center in an isotropic environment; in contrast, 19 equally spaced lines should be observed if the spin is delocalized over two magnetically equivalent niobium atoms. The experimental data for complex **2** is in between these two extremes, suggesting a more complex electronic structure.

The best simulation (Fig. 8) of the EPR data was obtained using two Nb atoms that have different hyperfine coupling constants (*A* ^93^Nb) and in a slight rhombic symmetry (*viz. g*_1_ = 1.980, *g*_2_ = 1.991, *g*_3_ = 1.997). Nb(1) has a large hyperfine coupling constant of 290 MHz (∼103 G) and two lower ones (150 and 100 MHz), while Nb(2) is isotropic (165 MHz, ∼59 G). No superhyperfine ^14^N coupling constants were used because the line shape is significantly broadened. Hybrid DFT-based G matrix and coupling constant calculations were carried out with the ORCA program employing two different functionals (B3LYP and PBE0). The results are in very good agreement with the hyperfine coupling constants obtained from the EPR experimental results (Table 2).

**Table 2:** Principal experimental and calculated EPR data for complex **1** (hyperfine coupling constants, *A*, are given in MHz)

	*g*_1_, *g*_2_, *g*_3_	*A* ^93^Nb(1)	*A* ^93^Nb(2)
Exp	1.980, 1.991, 1.997	100, 150, 290	165, 165, 165
PBE0	1.979, 1.991, 1.997	–125, –130, –295	171, 171, 187
B3LYP	1.979, 1.992, 2.000	–91, –90, –264	191, 174, 175

Therefore, the EPR data reinforce the bonding framework provided by the DFT calculations and spectroscopic data provided above, which can be summarized as follows. Complex **2** can be viewed as a mixed valence complex, in which Nb(1) carries the single electron in a predominantly metal based orbital that is partially delocalized through δ-donation into the formally empty benzene π orbitals of e_2u_ symmetry (using the notation for the D_6h_ symmetry of free benzene), leading to a lower isotropic coupling constant for Nb(2). Thus, Nb(2) carries two electrons in a 4d-orbital that interacts in a π-donating manner with the same set of formally empty benzene orbitals. From UV-visible, EPR and DFT calculations, we suggest that the unpaired electron is only partially delocalized from the Nb(1) into an empty d orbital of Nb(2) through the other (empty) e_2u_ orbital of the benzene moiety.

### Reactivity

**Scheme 2:**
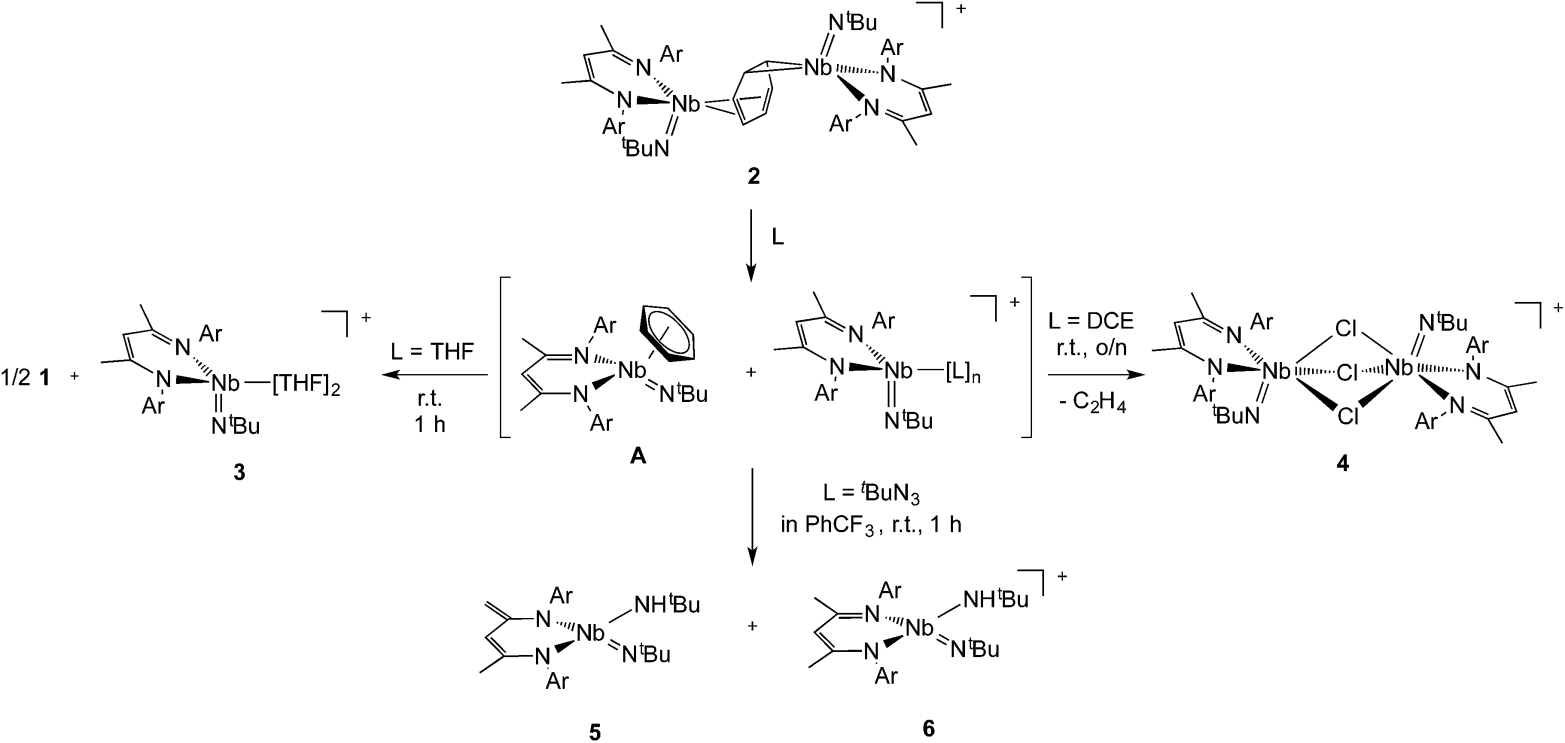


Interestingly, unlike the neutral sandwich complex **1** – which is both chemically and thermally stable – mixed-valence **2** reacted readily with coordinating solvents and halocarbons at room temperature. Dissolution of **2** in THF resulted in a fast exothermic reaction and a color change from deep green to red within 10 minutes. Removal of volatile materials resulted in a red-brown residue which was dissolved in hexanes, leaving an insoluble green powder. Filtration of the hexanes solution and cooling to –40 °C provided red crystals of complex **1**. The remaining green powder is highly unstable but could be crystallized by dissolution in THF and slow diffusion of hexanes at room temperature; this species was identified by X-ray diffraction as the Nb(iv) complex, {Nb(BDI)N^*t*^Bu(thf)_2_}{B(C_6_F_5_)_4_}, **3**{B(C_6_F_5_)_4_}. Such reactivity is consistent with the proposed mechanism in Scheme 2.

Coordination of the solvent to the electron deficient Nb(iv) metal center of the μ-benzene complex followed by dissociation results in the formation of the reactive new Nb(iv) cationic species {Nb(BDI)N^*t*^Bu(thf)_2_}^+^, **3**, and the monomeric benzene complex, Nb(BDI)N^*t*^Bu(C_6_H_6_);^24^ in solution at room temperature, the latter is known to decompose to form **1**.

**Fig. 9:**
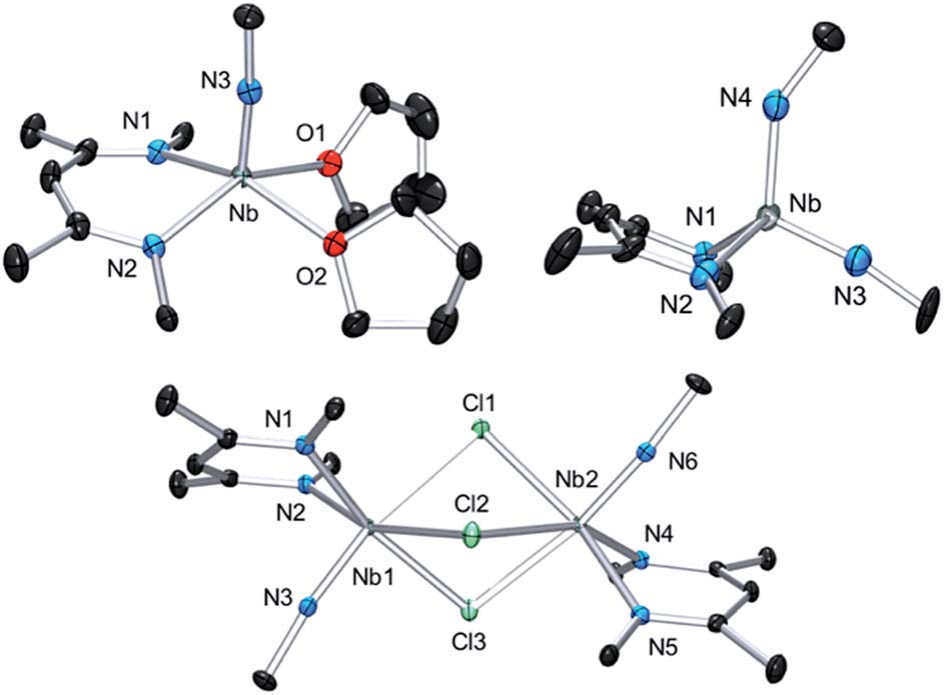
Thermal ellipsoid plot of **3** (top left), **4** (bottom) and **6** (top right). iPr_2_Ar groups of BDI ligand and CH_3_ groups of ^*t*^BuN ligand, counter ion {B(C_6_F_5_)_4_}^–^ and hydrogens have been removed for clarity. Thermal ellipsoids are represented at 50%. Crystallographic parameters are presented in Table S1.†

Complex **3** is a rare example of a monomeric d^1^ cationic niobium species stabilized by relatively labile ligands (two THF molecules). Crystallographic analysis of **3** (Fig. 9, top left) reveals the presence of a slightly distorted square planar pyramidal geometry (*τ* = 0.12), with the ^*t*^Bu imido group in the apical position along with the BDI and the two THF ligands in the basal plane. The metal nitrogen bond distances of the imido and BDI ligands are within previously described distances for such a ligand platform^43–45^ (Nb–N(3) = 1.7531(19) Å and Nb–N_BDI average_ = 2.182 Å), as well as the niobium oxygen bond distances (Nb–O_average_ = 2.221 Å).

Dissolution of **2** in 1,2-dichloroethane (DCE) at room temperature overnight results in colour change from deep green to orange from which crystals of {[Nb(BDI)N^*t*^Bu]_2_(μ-Cl)_3_}{B(C_6_F_5_)_4_}, **4**, were obtained in good yield (78%). The X-ray structure of **4** consists of a dimeric, cationic molecule in which the two Nb(BDI)N^*t*^Bu moieties are bridged by three chloride ligands (Fig. 9, bottom). The long Nb(1)–Nb(2) distance of 3.625 Å supports the absence of a metal–metal interaction, consistent with a d^0^/d^0^ electronic configuration of **4**. The steric hindrance imposed by the BDI ligand results in a non-symmetric coordination of the three chlorides. Thus, Cl(1) aligns with the BDI of Nb(1) and the ^*t*^Bu imido of Nb(2) and is closer to the Nb(2) compared to the other metal center (Cl(1)–Nb(1) = 2.6787(5) Å and Cl(1)–Nb(1) = 2.5119(4) Å). Likewise, Cl(3) aligns with the BDI of Nb(2) and the ^*t*^Bu imido of Nb(1) (Cl(3)–Nb(1) = 2.5249(5) Å and Cl(3)–Nb(2) = 2.6963(5) Å). Finally, the third chloride Cl(3) is staggered with respect to both BDI ligands and coordinates symmetrically to the two metal centers (Cl(2)–Nb(1) = 2.5559(4) Å and Cl(2)–Nb(1) = 2.5567(4) Å). NMR spectroscopic characterization of this diamagnetic Nb(v)/Nb(v) complex indicates that **4** is highly symmetric in solution. *In situ* ^1^H NMR spectroscopy studies of the reaction between **2** and DCE revealed the clean formation of **4** and ethane. Formation of **4** from **2** was also observed in CDCl_3_ with d_2_-tetrachloroethane being generated as a side product, albeit at a much slower rate (80 °C, 24 h). The number of chlorines abstracted from DCE (or CDCl_3_) and the formation of ethylene (or C_2_D_2_Cl_4_) is in agreement with the presence of three Nb d-based electrons in **2**.

Finally, addition of excess ^*t*^BuN_3_ (5 equiv.) to a solution of **2** in α,α,α-trifluorotoluene results in a fast colour change from green to orange along with vigorous gas evolution. Extraction with toluene yielded [(CH_2_C(NAr)CHC(NAr)CH_3_)Nb(N^*t*^Bu)(NH^*t*^Bu)], **5**, which has been reported previously.^44^ Extraction of the remaining yellow residue with dichloromethane followed by crystallization at –40 °C yielded X-ray quality single crystals, which were formulated as a new cationic complex {Nb(BDI)(N^*t*^Bu)(NH^*t*^Bu)}{B(C_6_F_5_)_4_}, **6**, by single-crystal X-ray diffraction (Fig. 9, top right). The structure indicates a tetrahedral coordination environment at Nb with two inequivalent ^*t*^BuN moieties, consistent with an imido amide complex (Nb–N(3) = 1.733(3) Å and Nb–N(4) = 1.953(4) Å; Nb–N(3)–C(30) = 172.4° and Nb–N(4)–C(34) = 146.4°). Interestingly, as observed in complex **2**, the BDI backbone is distorted toward the metal centre; however, unlike **2**, the metal center appears to be in close contact with the C_alpha_ of the imine (Nb–C(4) = 2.694(4) Å), and suggests the presence of an η^2^-imine coordination of one side of BDI to the coordinatively unsaturated and electron poor metal centre. Complex **5** was previously isolated as a tautomerization product of the bis *tert*-butyl imido niobium BDI complex, which was synthesized either from the reaction of Nb(BDI)(N^*t*^Bu)Cl_2_ with two equivalents of ^*t*^BuNHLi,^44^ or *via* reaction between a trivalent niobium BDI imido moiety, such as the mono arene niobium complex **A**, and ^*t*^BuN_3_.^46^ Overall, these reactions are in good agreement with the electronic structure set out above and emphasizes well the different nature of the two Nb atoms, both stabilized by the benzene ring.

## Conclusions

Our results show that exploring the redox chemistry of bimetallic arene complexes leads to reactivity that was inaccessible from neutral precursors. In the symmetric parent complex **1**, the calculations indicate that the HOMO and HOMO-1 are δ-backbonding between the niobiums and the bridging arene ligand. Solution and solid-state structural characterization indicates that reaction of complex **1** with Ag{B(C_6_F_5_)_4_} resulted in a one-electron oxidation that destabilizes the HOMO and disrupts the symmetry to produce a new cationic complex **2**. Nb L_3,2_-edge XANES measurements indicate that **2** has a mixed-valence electronic structure, and that the best integral valence model is of a (4d^1^)(4d^2^) complex with a neutral arene ligand. UV-visible and EPR spectroscopy also support this picture. However, the results also suggest that electron delocalization into the empty benzene orbitals of e_2u_ symmetry from both Nb atoms occurs, resulting in partial population of these orbitals without resulting in complete reduction of the arene. This suggests that oxidation state formalisms are inappropriate metrics of electronic structure in bimetallic arene complexes. Finally, whereas **1** was found to be inert in terms of chemical reactivity, the cation **2** possesses two chemically inequivalent niobium atoms that afford access to metal-based chemistry with relatively unreactive substrates.

## Experimental section

### General considerations

Unless otherwise noted, all reactions were performed either using standard Schlenk line techniques or in an MBraun inert atmosphere glove box under an atmosphere of purified nitrogen (<1 ppm O_2_/H_2_O). Glassware, cannulae, and Celite® were stored in an oven at *ca.* 160 °C for at least 12 h prior to use. *n*-Pentane, hexanes, Et_2_O, THF, toluene and benzene were purified by passage through a column of activated alumina, stored over 3 or 4 Å molecular sieves, and degassed prior to use.^47^ α,α,α-Trifluorotoluene, 1,2-dichloroethane and chlorobenzene were dried over P_2_O_5_, distilled under reduced pressure, degassed and stored over 4 Å molecular sieves. Deuterated solvents (C_6_D_6_, C_7_D_8_ and C_6_D_12_) were dried over sodium/benzophenone, and C_6_D_5_Cl was dried over CaH_2_. The deuterated solvents were then vacuum transferred to a storage flask and degassed before being stored in the dry box. C_6_D_6_, C_7_D_8_ and C_6_D_5_Cl were stored over activated molecular sieves. *N*,*N*′-bis-(2,6-diisopropylphenyl)-β-diketiminate (BDI),^48^ Li(BDI)·Et_2_O,^49^ (BDI)pyCl_2_Nb(N^*t*^Bu)^50^ (complex **7**) and (BDI)(Me)_2_Nb(N^*t*^Bu)^50^ were prepared using literature procedures. All other reagents were acquired from commercial sources and used as received. NMR spectra were recorded on Bruker AV-300, AVQ-400, AVB-400, DRX-500, AV-500, and AV-600 spectrometers. Chemical shifts were measured relative to residual solvent peaks, which were assigned relative to an external TMS standard set at 0.00 ppm. ^1^H and ^13^C NMR assignments were routinely confirmed by ^1^H–^1^H (COSY, NOESY) and ^1^H–^13^C (HSQC and HMBC) experiments. Samples for UV-vis-NIR spectroscopy were prepared in a Schlenk-adapted quartz cuvette and analyzed on a Varian Cary 50 scanning spectrophotometer. The uncorrected melting points were determined using sealed capillaries prepared under nitrogen on an Optmelt SRS. Elemental analyses were performed at the College of Chemistry Microanalytical Laboratory, University of California, Berkeley. The X-ray structural determinations were performed at CHEXRAY, University of California, Berkeley on Bruker SMART 1000 or SMART APEX diffractometers.

### {[Nb(BDI)N^*t*^Bu]_2_(μ-C_6_H_6_)}{B(C_6_F_5_)_4_} (**2**)

A solution of Ag[B(C_6_F_6_)_4_] (384 mg, 0.488 mmol, 1.05 equiv.) in 20 mL of Et_2_O was slowly added to a solution of complex **1** (616 mg, 0.465 mmol, 1 equiv.) in 100 mL of hexanes at 0 °C in the dark. The red mixture, which quickly turned dark green, was stirred at 0 °C for an hour and then at room temperature for another hour from which a large quantity of green microcrystalline powder formed along with black silver. After removal of the volatile material under reduced pressure and washing with 2 × 20 mL of Et_2_O, the green material was extracted with α,α,α-trifluorotoluene and crystallized at –20 °C. Dark green crystals of complex **2** were thus obtained in good yield (585 mg, 65%). X-ray quality crystals were obtained from recrystallization in DCE at –20 °C, however **2** was found to slowly react at room temperature with DCE. ^1^H NMR (500 MHz, CDCl_3_, 293 K): 9.4 (2H), 6.88 (2H), 4.57 (2H), 3.12 (7H), 1.11 (24H), –0.24 (9H), –0.95 (4H). ^19^F NMR (470 MHz, CDCl_3_, 293 K): *δ* (ppm) –131.59, –163.14, –166.80 ([B(C_6_F_6_)_4_]^–^). Anal. calcd for C_98_H_110_B_1_Cl_2_F_20_N_6_Nb_2_: C, 58.29; H, 5.49; N, 4.16. Found: C, 58.19; H, 5.55; N, 4.16.

### {[Nb(BDI)N^*t*^Bu]_2_(μ-C_6_D_6_)}{B(C_6_F_5_)_4_} (**2-d_6_**)

A solution of AgB(C_6_F_6_)_4_ (30 mg, 0.04 mmol, 1.05 equiv.) in 5 mL of Et_2_O was slowly added to a solution of complex **1-d_6_** (60 mg, 0.04 mmol, 1 equiv.) in 10 mL of hexanes at 0 °C in the dark. The reaction mixture was worked up as described in the synthesis of **2**. Dark green crystals of complex **2-d_6_** were obtained in good yield (51 mg, 62%). The ^1^H and ^19^F NMR spectra were similar to those of complex **2**.

### {Nb(BDI)(N^*t*^Bu)(THF)_2_}{B(C_6_F_5_)_4_} (**3**)

Complex **2** (300 mg, 0.013 mmol) was dissolved in 50 mL of THF, the solution turned rapidly from deep green to red-orange. After stirring at room temperature for 12 hours, the volatiles were removed under reduced pressure resulting in a brown powder. The complex **1** was extracted and crystallized at –40 °C from hexanes affording deep red crystals in moderate yield (95 mg, 42%). ^1^H and ^13^C NMR spectroscopic analysis of **1** are in agreement with the data reported in the literature.^24^ The residual green powder was extracted with THF, and crystals were obtained from hexanes layering at room temperature. ^19^F NMR (470 MHz, CDCl_3_, 293 K): *δ* (ppm) –131.59, –163.14, –166.80 ([B(C_6_F_6_)_4_]^–^). Anal. calcd for C_65_H_66_B_1_F_20_N_3_Nb: C, 55.57; H, 4.74; N, 2.99. Found: C, 55.19; H, 5.12; N, 3.25.

### {[Nb(BDI)(N^*t*^Bu)]_2_(μ-Cl)_3_}{B(C_6_F_5_)_4_} (**4**)

Complex **2** (500 mg, 0.24 mmol) was dissolved in 30 mL of DCE, and the solution turned slowly from deep green to bright orange. After being stirred at room temperature overnight, the solution was concentrated and stored at –20 °C for two days, resulting in the crystallization of **3** as orange crystals in good yield (410 mg, 82%). X-ray quality crystals were obtained by recrystallization from DCE at 0 °C. ^1^H NMR (500 MHz, CDCl_3_, 293 K): 7.31 (t, 2H, *p*-Ar, ^3^ *J*_HH_ = 7.6 Hz), 7.24 (dd, 2H, *o*-Ar), 7.10 (dd, 2H, *o*-Ar), 6.34 (s, 1H, HC(C(Me)NAr)_2_), 3.10 (sept, 2H, CHMe_2_, ^3^ *J*_HH_ = 6.8 Hz), 2.52 (sept, 2H, CHMe_2_, ^3^ *J*_HH_ = 6.9 Hz), 2.04 (s, 6H, HC(C(Me)NAr)_2_), 1.31 (d, 6H, CHMe_2_, ^3^ *J*_HH_ = 6.8 Hz), 1.22 (s, 9H, Nb=N^*t*^Bu), 1.14 (d, 6H, CHMe_2_, ^3^ *J*_HH_ = 6.6 Hz), 1.05 (d, 6H, CHMe_2_,^3^ *J*_HH_ = 6.9 Hz), 0.61 (m, 6H, CHMe_2_, ^3^ *J*_HH_ = 6.9 Hz). ^13^C NMR (100 MHz, CDCl_3_, 293 K): 172.5 (C, HC(C(Me)NAr)_2_), 144.1 (C, Ar), 140.5 (C, Ar), 140.2 (C, Ar), 127.4 (CH, Ar), 125.6 (CH, Ar), 124.1 (CH, Ar), 65.9 (CH, HC(C(Me)NAr)_2_), 30.4 (CH_3_, Nb=N^*t*^Bu, C_α_), 31.4 (CH, CHMe_2_ of C=NAr), 31.0 (CH_3_, Nb=NH^*t*^Bu, C_β_), 29.7 (CH_3_, HC(C(Me)NAr)_2_), 28.9 (CH, CHMe_2_ of C=NAr), 24.1 (CH_3_, CHMe_2_ of C=NAr), 23.8 (CH_3_, CHMe_2_ of C=NAr), 23.6 (CH_3_, CHMe_2_ of C=NAr), 22.6 (CH_3_, CHMe_2_ of C=NAr). ^19^F NMR (470 MHz, CDCl_3_, 293 K): *δ* (ppm) –131.59, –163.14, –166.80 ([B(C_6_F_6_)_4_]^–^). Anal. calcd for C_90_H_100_B_1_Cl_3_F_20_N_6_Nb_2_: C, 55.47; H, 5.17; N, 4.31. Found: C, 55.15; H, 5.45; N, 4.06.

### Nb(BDI^#^)(N^*t*^Bu)(NH^*t*^Bu) (**5**) and {Nb(BDI)(N^*t*^Bu)(NH^*t*^Bu)}{B(C_6_F_5_)_4_} (**6**)

Complex **2** (170 mg, 0.08 mmol) was dissolved in 50 mL of α,α,α-trifluorotoluene, a dilute solution of ^*t*^BuN_3_ in α,α,α-trifluorotoluene (40 mg, 0.4 mmol in 5 mL of solvent) was added dropwise and the solution turned rapidly from deep green to light yellow with strong gas evolution. After being stirred at room temperature for 2 hours, the volatile material was removed under reduced pressure. The complex **5**, [(BDI^#^)Nb(N^*t*^Bu)(NH^*t*^Bu)] (with BDI^#^ =H_2_C=C(NAr)CH=C(NAr)Me), was extracted and crystallized at –40 °C from toluene affording orange crystals in moderate yield (54%). ^1^H and ^13^C NMR spectroscopic analysis are in agreement with the complex **5** previously reported in the literature.^44^ Complex **6**, [(BDI)Nb(N^*t*^Bu)(NH^*t*^Bu)][B(C_6_F_5_)_4_], was extracted and crystallized at –40 °C from dichloromethane affording colorless crystals in good yield (90 mg, 85%). X-ray quality crystals of complex **6** were obtained by recrystallization from a DCM/hexanes layering at room temperature. ^1^H NMR (500 MHz, CDCl_3_, 293 K): 7.38 (t, 2H, *p*-Ar, ^3^ *J*_HH_ = 7.6 Hz), 7.30 (m, 4H, *m*- and *o*-Ar), 6.17 (s, 1H, HC(C(Me)NAr)_2_), 2.60 (sept, 2H, CHMe_2_, ^3^ *J*_HH_ = 6.6 Hz), 2.43 (sept, 2H, CHMe_2_, ^3^ *J*_HH_ = 6.9 Hz), 1.73 (s, 6H, HC(C(Me)NAr)_2_), 1.37 (d, 6H, CHMe_2_, ^3^ *J*_HH_ = 6.6 Hz), 1.30 (s, 9H, Nb=N^*t*^Bu), 1.24 (d, 6H, CHMe_2_, ^3^ *J*_HH_ = 6.6 Hz), 1.17 (d, 6H, CHMe_2_,^3^ *J*_HH_ = 6.9 Hz), 1.09 (m, 6H, CHMe_2_, ^3^ *J*_HH_ = 6.9 Hz), 0.87 (s, 9H, Nb=NH^*t*^Bu). ^13^C NMR (100 MHz, CDCl_3_, 293 K): 171.5 (C, HC(C(Me)NAr)_2_), 142.4 (C, Ar), 140.9 (C, Ar), 140.2 (C, Ar), 129.4 (CH, Ar), 125.6 (CH, Ar), 124.6 (CH, Ar), 65.9 (CH, HC(C(Me)NAr)_2_), 32.4 (CH_3_, Nb=N^*t*^Bu, C_α_), 31.9 (CH, CHMe_2_ of C=NAr), 31.3 (CH_3_, Nb=NH^*t*^Bu, C_β_), 29.9 (CH_3_, HC(C(Me)NAr)_2_), 28.2 (CH, CHMe_2_ of C=NAr), 24.9 (CH_3_, CHMe_2_ of C=NAr), 24.7 (CH_3_, CHMe_2_ of C=NAr), 24.6 (CH_3_, CHMe_2_ of C=NAr), 24.6 (CH_3_, CHMe_2_ of C=NAr), 15.4 (C, Nb=N^*t*^Bu, C_α_), 14.1 (C, Nb=NH^*t*^Bu, C_α_). ^19^F NMR (470 MHz, CDCl_3_, 293 K): *δ* (ppm) –131.59, –163.14, –166.80 ([B(C_6_F_6_)_4_]^–^). Anal. calcd for C_61_H_60_B_1_F_20_N_4_Nb_1_: C, 54.97; H, 4.54; N, 4.20. Found: C, 54.89; H, 4.55; N, 4.35.

### Cyclic voltammetry

Electrochemical measurements were obtained with a BASi Epsilon potentiostat at room temperature using a glassy carbon working electrode, a platinum counter electrode, and a silver wire floating reference electrode. Cyclic voltammograms were recorded in a glovebox at room temperature in α,α,α-trifluorotoluene solution containing 0.1 M [^*n*^Bu_4_N][BF_4_] as the supporting electrolyte and 0.001 M of either **1** or **2**. All potentials were referenced against a [Cp_2_Co]^0/+^ internal standard, which were then standardized against both [Cp_2_Fe]^0/+^ and Ag/Ag^+^ (*E*_1/2_ [Cp_2_Co]^0/+^ = –1.3 V *vs.* [Cp_2_Fe]^0/+^ and *E*_1/2_ [Cp_2_Co]^0/+^ = –0.85 V *vs.* Ag/Ag^+^).

### Crystallographic analysis

X-ray structural determinations were performed on a Bruker SMART 1000 or SMART APEX diffractometer. Both are 3-circle diffractometers that couple a CCD detector^51^ with a sealed-tube source of monochromated Mo Ka radiation (*λ* = 0.71073 Å). A crystal of appropriate size was coated in Paratone-N^®^ oil and mounted on a Kapton^®^ loop. The loop was transferred to the diffractometer, centered in the beam, and cooled by a nitrogen flow low-temperature apparatus that had been previously calibrated by a thermocouple placed at the same position as the crystal. Preliminary orientation matrices and cell constants were determined by collection of 60 10 s frames, followed by spot integration and least-squares refinement. The reported cell dimensions were calculated from all reflections with *I* > 10*σ*. The data were corrected for Lorentz and polarization effects; no correction for crystal decay was applied. An empirical absorption correction based on comparison of redundant and equivalent reflections was applied using SADABS.^52^ All software used for diffraction data processing and crystal-structure solution and refinement are contained in the APEX2 program suite (Bruker AXS, Madison, WI).^53^ Thermal parameters for all non-hydrogen atoms were refined anisotropically. For all structures, *R*_1_ = Σ(|*F*_o_| – |*F*_c_|)/Σ(|*F*_o_|); w*R*_2_ = [Σ{w(*F*_o_^2^ – *F*_c_^2^)^2^}/Σ{w(*F*_o_^2^)^2^}]^1/2^. Thermal ellipsoid plots were created using the ORTEP-3 software package and POV-ray.^54^

### Magnetic susceptibility and EPR spectroscopy

Magnetic susceptibility measurements were made for complex **2** at 10 and 40 kOe in a 7 T Quantum Design Magnetic Properties Measurement System, that utilized a superconducting quantum interference device (SQUID). The sample was contained in sealed quartz tube. EPR spectra were obtained with a Varian E-12 spectrometer, an EIP-547 microwave frequency counter, and a Varian E-500 gaussmeter, which was calibrated using 2,2-diphenyl-1-picrylhydrazyl (DPPH, *g* = 2.0036). The simulated spectra were obtained using EasySpin.^55^

### Nb L_3,2_-edge XANES measurements

Niobium L_3,2_-edge XANES spectra were acquired using the STXM instrument at the Advanced Light Source beamline 5.3.2.2. Sample preparation methodology, data collection and analysis procedures were as described in an earlier publication.^24^

### DFT calculations

Gradient-corrected DFT calculations were carried out using the PBE functional,^56,57^ as implemented in the Amsterdam Density Functional 2012.01 (ref. 58 and 59) (ADF) quantum chemistry code. Scalar relativistic effects were incorporated using the Zeroth Order Regular Approximation (ZORA) Hamiltonian. A Slater Type Orbital ZORA basis set of TZP quality was used for Nb, with all other atoms being treated with a DZP ZORA basis. For geometry optimizations, the frozen core approximation was employed; Nb(3d), C(1s), N(1s). The default SCF and geometry convergence criteria were used, together with an integration grid of 4.5. The EPR g matrix, hyperfine coupling constants and TD-DFT calculations were obtained from single point calculation, on the calculated optimized geometry obtained starting from the X-ray crystal structure data and that was described in the DFT section, using the ORCA program package.^60^ Two hybrid functionals B3LYP^61,62^ and PBE0 were employed. The TZVP^63^ basis set was used for all carbon and hydrogen atoms, while a TZVPP^64^ basis set was used for the niobium atoms and an IGLO-III basis set for the nitrogen atoms for the EPR g matrix and hyperfine coupling calculations while the TZVP basis sets have been used for the nitrogen atoms for the TD-DFT calculations.^65^ For all calculations integration grids (Grid4, ORCA convention) and tight SCF convergence were used. Scalar relativistic corrections were included in the valence space *via* the ZORA formalism.
