# Supplementary material for: Electron localization in a mixed-valence diniobium benzene complex
Source: Chem Sci. 2014 Nov 11;6(2):993–1003. doi: 10.1039/c4sc02705a (PMC5811154; doi:10.1039/c4sc02705a)
Supplement: Supplementary file 1 [file SC-006-C4SC02705A-s001.pdf]

Electronic Supplementary Material (ESI) for Chemical Science.
This journal is © The Royal Society of Chemistry 2014

Electron Localization in a Mixed-ValenceDiniobium Benzene Complex

Thomas L. Gianetti,[a] Grégory Nocton,[b]* Stefan G. Minasian,[c,d] Nikolas Kaltsoyannis,[e]* A. L. David Kilcoyne,[c] Stosh A. Kozimor,[d] David K. Shuh,[c] Tolek Tyliszczak,[f] Robert G. Bergman[a]* and John Arnold.[a]*

a. Department of Chemistry, University of California, Berkeley, CA94720
b. Laboratoire de Chimie Moléculaire, CNRS, Ecole Polytechnique, 91128 Palaiseau, France
c. Chemical Sciences Division, Lawrence Berkeley National Laboratory, Berkeley, CA 94720
d. Chemistry Division, Los Alamos National Laboratory, Los Alamos, NM 87545
e. Department of Chemistry, University College London, 20 Gordon Street, London, WC1H0AJ, UK
f. Advanced Light Source, Lawrence Berkeley National Laboratory, Berkeley, CA 94720

A. X-ray crystallography of 2, 3, 4, and 6 .... S.2
B. Cyclic voltammetry S.6
  B.1 Complex 1 .... S.6
  B.2 Complex 2 .... S.7
C. Spectroscopic analysis of 2 .... S.8
  C.1 NMR spectroscopy S.8
  C.2 Magnetism S.10
  C.3. Electron Paramagnetic Resonance (EPR) Spectroscopy S.11
D. DFT calculation S.12
  D.1. TD-DFT calculation S.12
  D.2. DFT optimized Cartesian atomic coordinates of 1 and 2. .... S.13

A. X-ray crystallography of 2, 3, 4, and 6

Table S1. Crystallographic parameters

**Compound**	**2.(DCE)**	**3**	**4.(2DCE)**	**6**
Formula	$C_{98}H_{110}B_1Cl_2F_{20}N_3Nb_1$	$C_{65}H_{66}B_1F_{20}N_3Nb_1O_2$	$C_{95}H_{110}B_1Cl_8F_{20}N_6Nb_2$	$C_{61}H_{59}B_1F_{20}N_3Nb_1$
Formula weight	2019.45	1404.93	2196.12	1331.84
Space Group	$P2_1$/c	$P2_1$/c	$P2_1$/c	*Pn*
a (Å)	14.224(3)	18.1669(5)	20.744(2)	15.2666(6)
b (Å)	21.737(3)	17.1279(5)	20.888(2)	16.9177(6)
c (Å)	31.247(2)	19.7092(5)	22.683(3)	25.5770(10)
α (°)	90	90	90	90
β (°)	90.83(4)	90.5590(10)	90.709(2)	97.658(2)
γ (°)	90	90	90	90
V ($Å^3$)	9660.2(2)	6132.4(3)	9827.6(19)	6547.0(4)
Z	4	4	4	4
ρ_{calcd} (g/cm^3)	1.389	1.522	1.484	1.351
F_{000}	4164	2876	4500	2716
μ (mm^{-1})	0.380	0.305	0.537	0.280
T_{min}/T_{max}	0.8508/0.9453	0.9140 /0.9415	0.8993/0.9892	0.9592 /0.9834
No. rflns measured	79928	57885	157286	66108
No. indep. rflns	17729	11263	17992	23491
R_{int}	0.0364	0.0347	0.0251	0.0483
No. obs. (I> 2.00σ(I))	17729	11263	17992	23491
No. variables	1228	842	1234	1599
R_1, wR_2	0.0562, 0.0929	0.0363, 0.0946	0.0257, 0.0621	0.0418, 0.0847
R_1 (all data)	0.0702	0.0458	0.0288	0.0559
GoF	1.056	1.022	1.058	1.016
Res. peak/hole ($e^-/Å^3$)	1.707/-1.093	0.774/-0.488	1.768/-0.665	0.436/-0.480

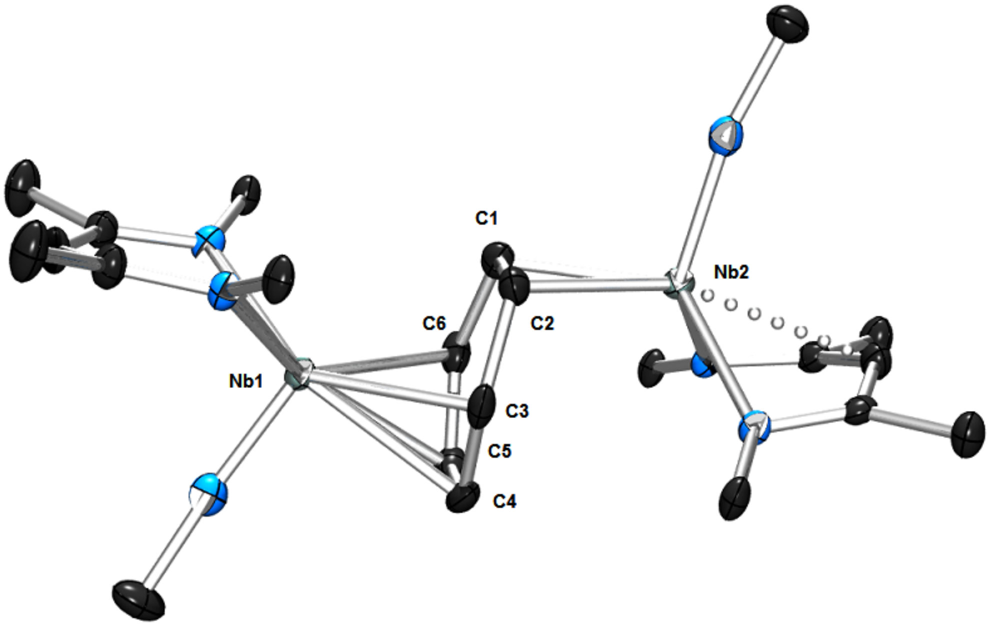

Figure S1. ORTEP diagram of **2**, hydrogens have been removed for clarity. Thermal ellipsoids are represented at 50 %.

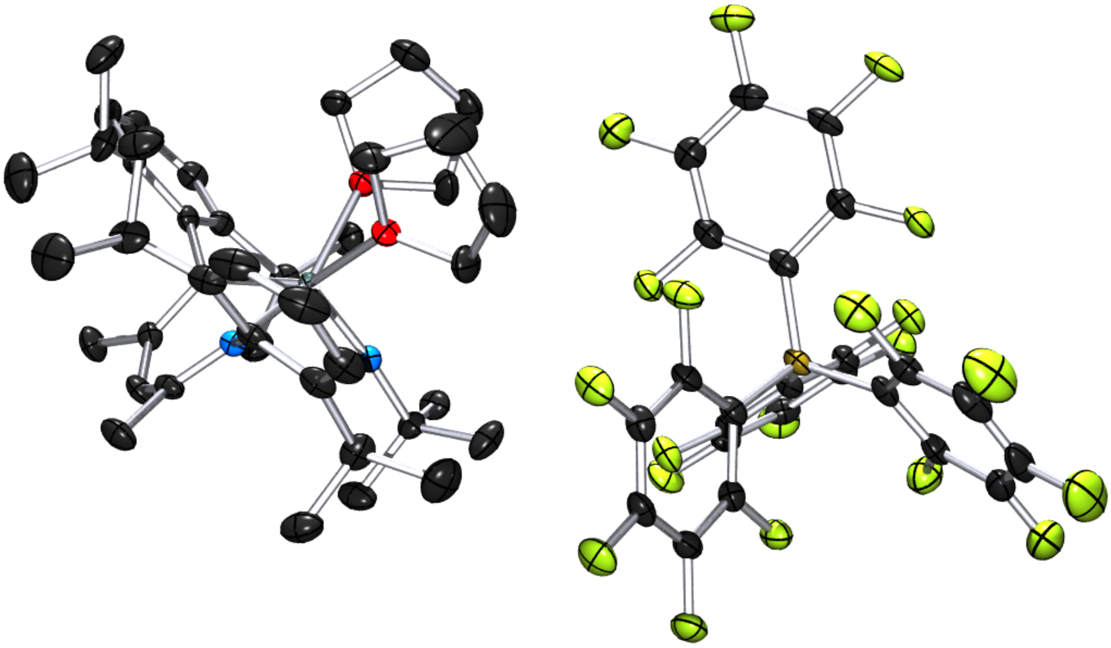

Figure S2. ORTEP diagram of **3**, hydrogens have been removed for clarity. Thermal ellipsoids are represented at 50 %.

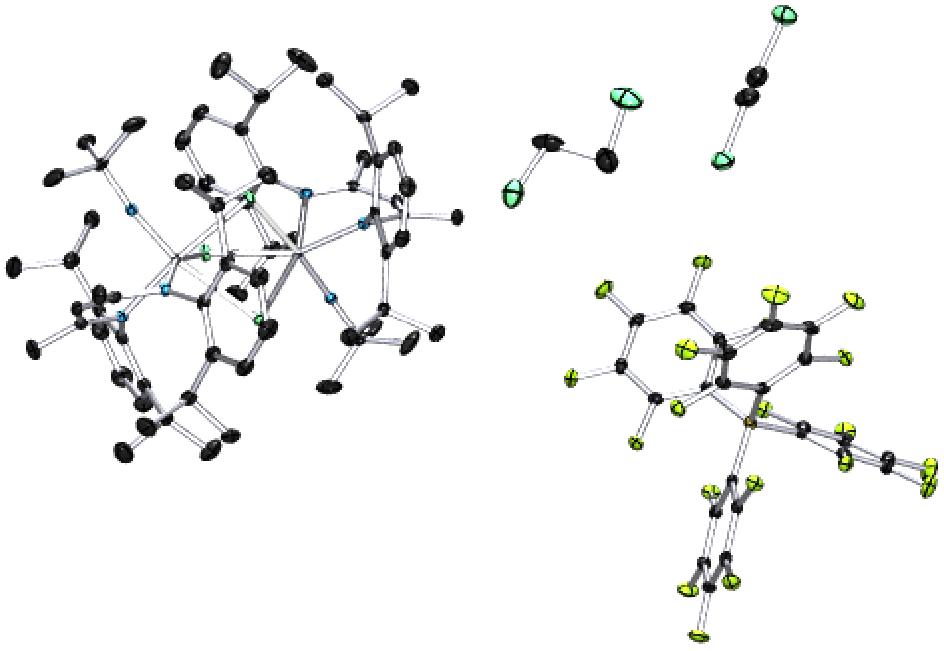

Figure S3. ORTEP diagram of **4**, hydrogens have been removed for clarity. Thermal ellipsoids are represented at 50 %.

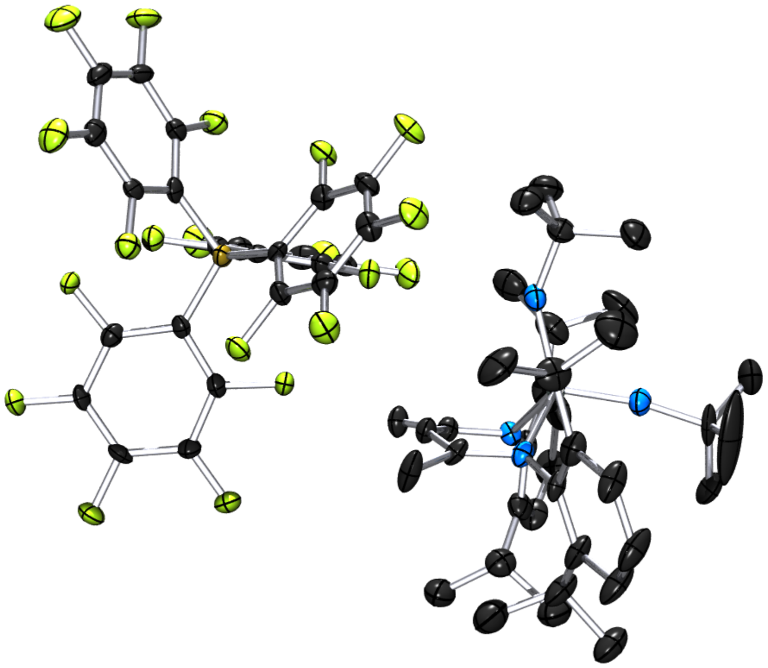

Figure S4. ORTEP diagram of **6**, hydrogens have been removed for clarity. Thermal ellipsoids are represented at 50 %.

B. Cyclic voltammetry

B.1 Complex 1

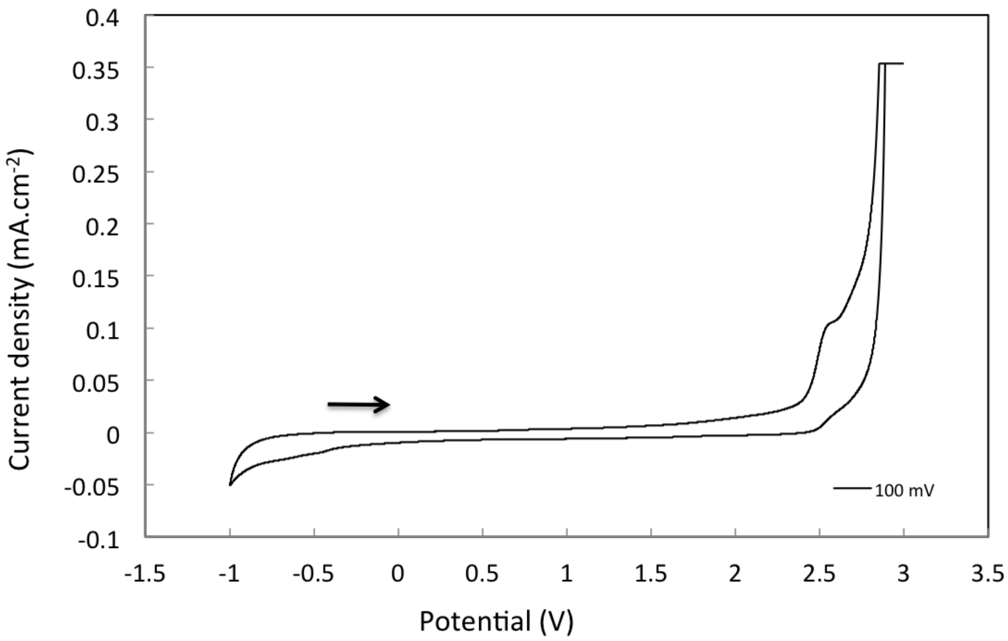

Figure S5 Background scan for the cyclic voltammetry of complex **1**

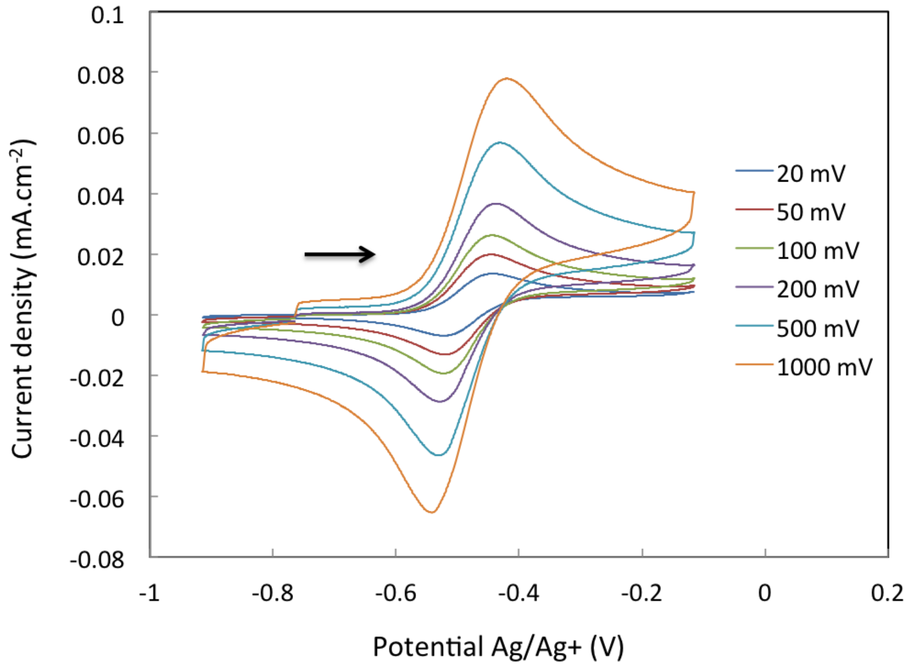

Figure S6 Cyclic voltammetry of complex **1** in α,α,α-trifluorotoluene at different scan-rate.

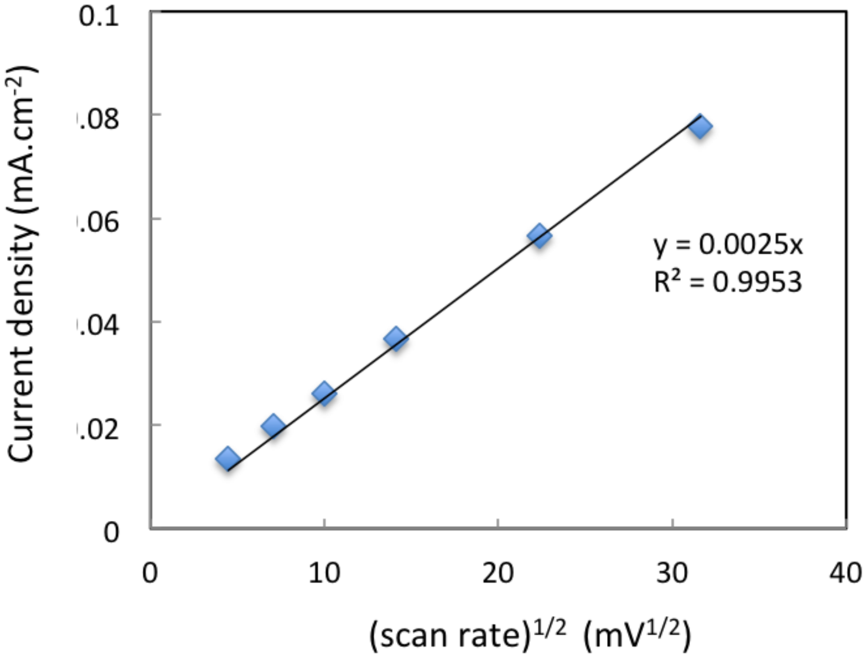

Figure S7 Plot of the peak current versus the square root of the scan rate of complex **1**

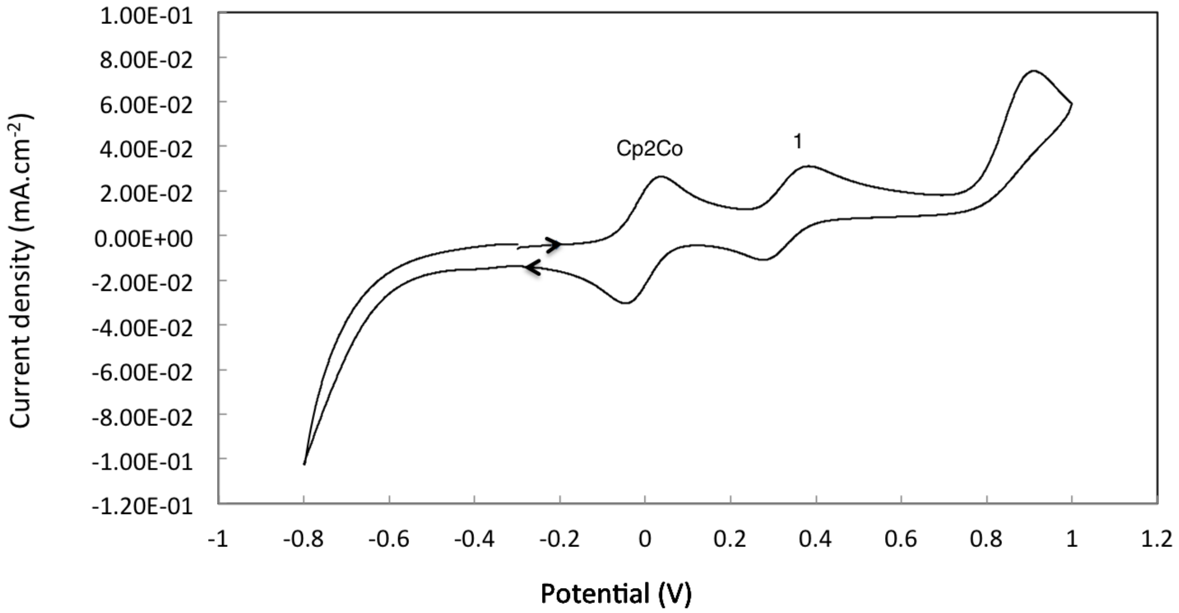

Figure S8 Cyclicvoltametry of complex **1** in α,α,α-trifluorotoluene with a scan rate of 100mV/s referenced to $[Cp_2Co]^{0/+}$ internal standard.

B.2 Complex 2

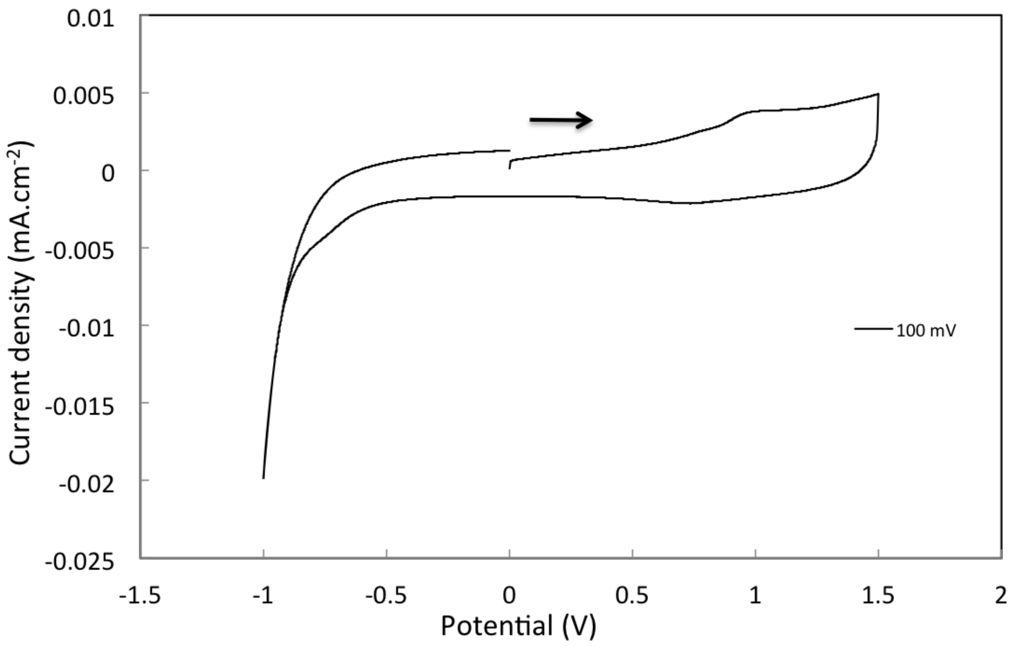

Figure S9 Background scan for the cyclic voltammetry of complex **2**

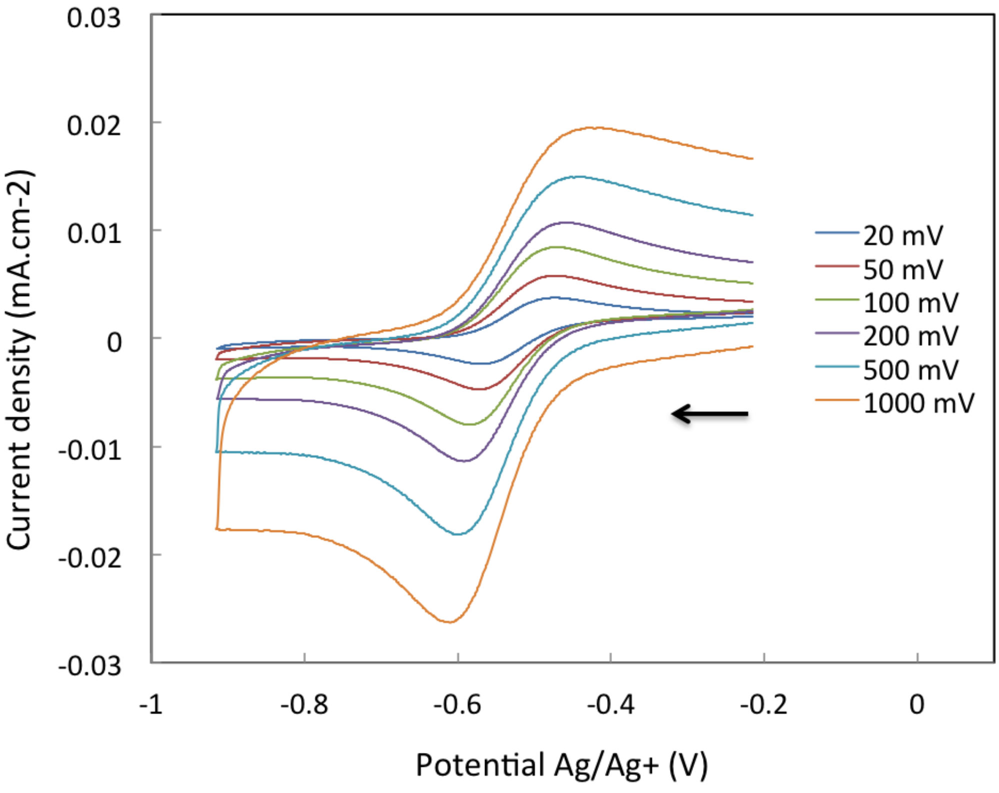

Figure S10 Cyclic voltammetry of complex **2** in α,α,α-trifluorotoluene at different scan-rate.

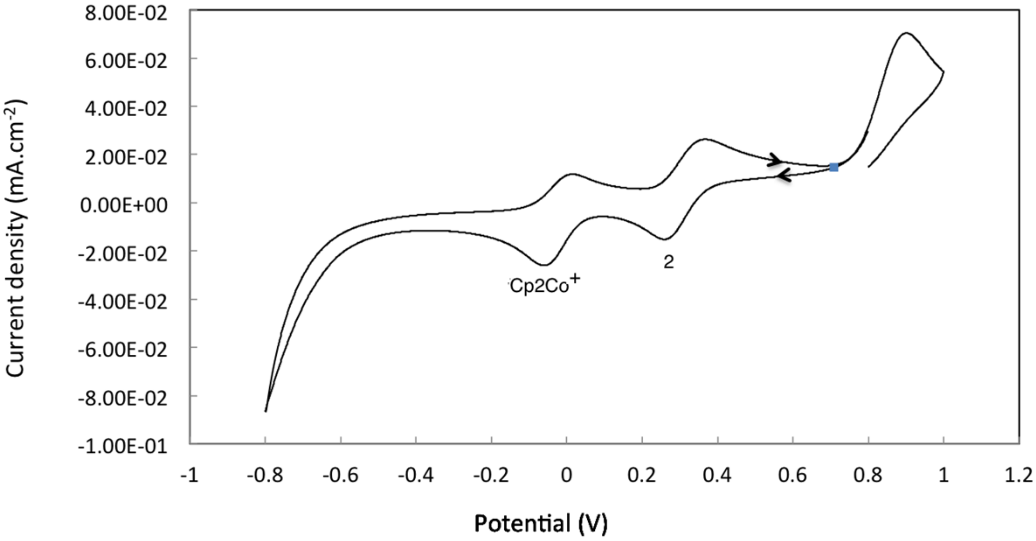

Figure S11 Cyclicvoltametry of complex **2** in α,α,α-trifluorotoluene with a scan rate of 100mV/s referenced to $[Cp_2Co]^{0/+}$ internal standard.

C. Spectroscopic analysis of 2

C.1 NMR spectroscopy

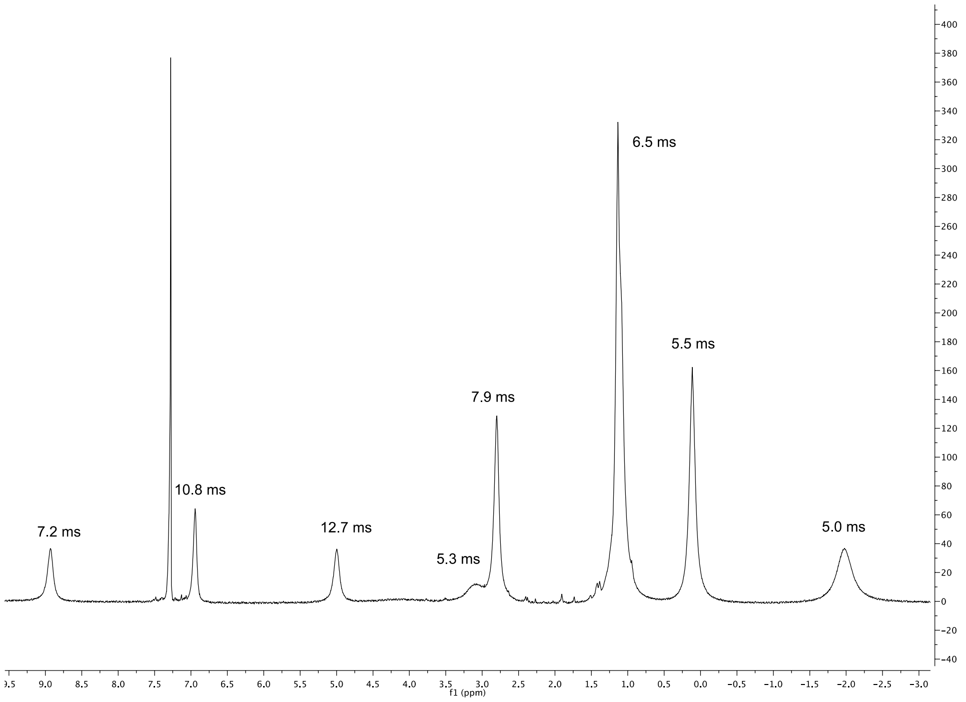

Figure S12. ^{1}H NMR spectrum of **2** and relaxation time of each resonanceat 350 K.

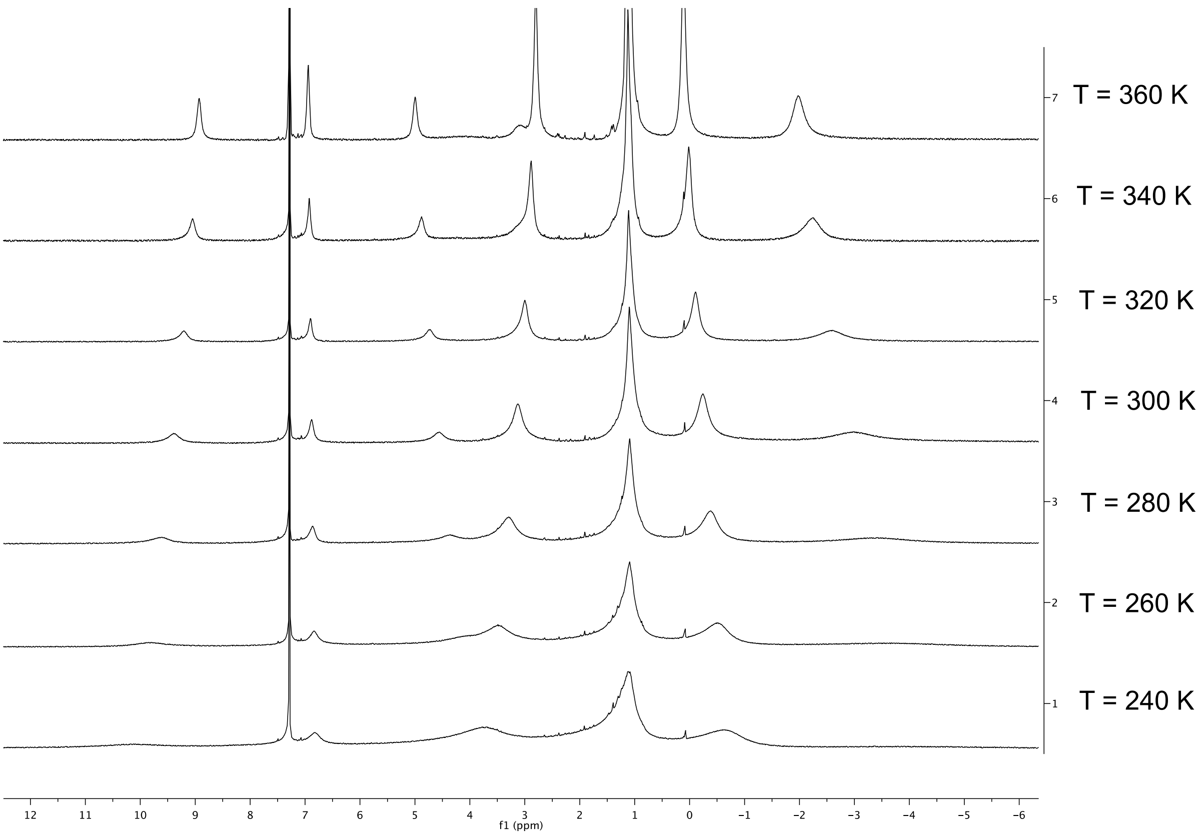

Figure S13. Variable temperature ^{1}H NMR spectroscopy of **2**

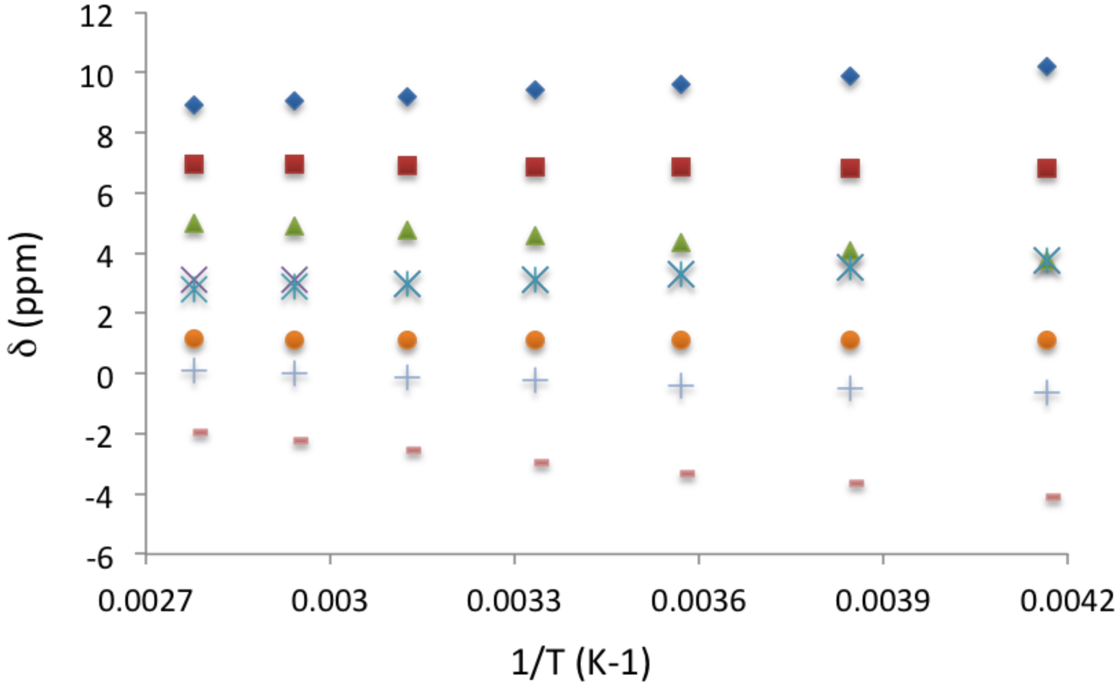

Figure S14. Temperature dependence of the chemical shifts of **2.**

C.2 Magnetism

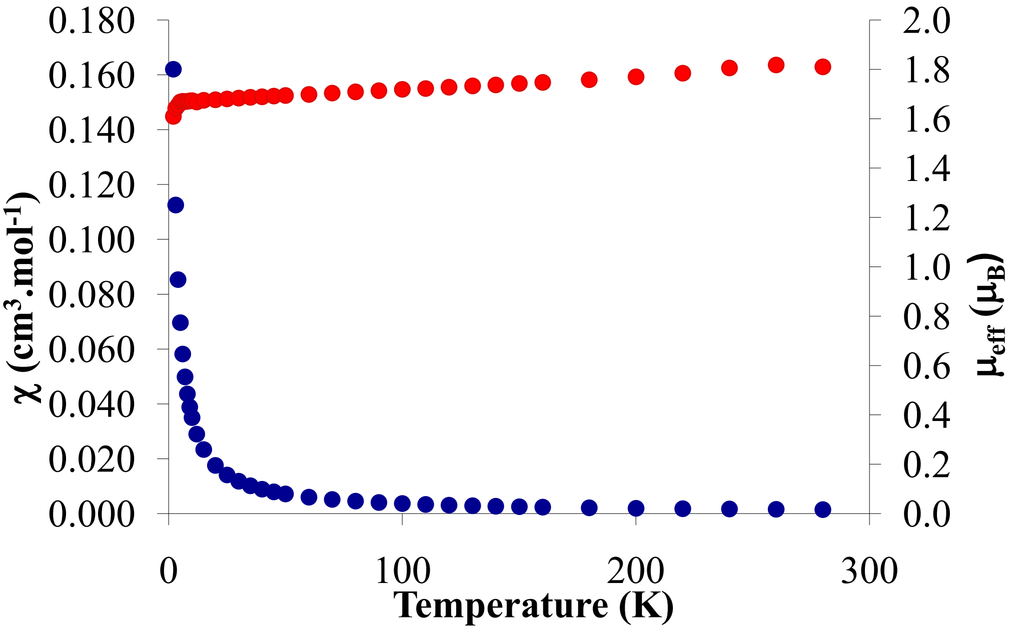

Figure S15. Temperature dependant magnetic data for **2.** Plot of χ vs. T (blue dots) and μ_{eff} vs. T (red dots).

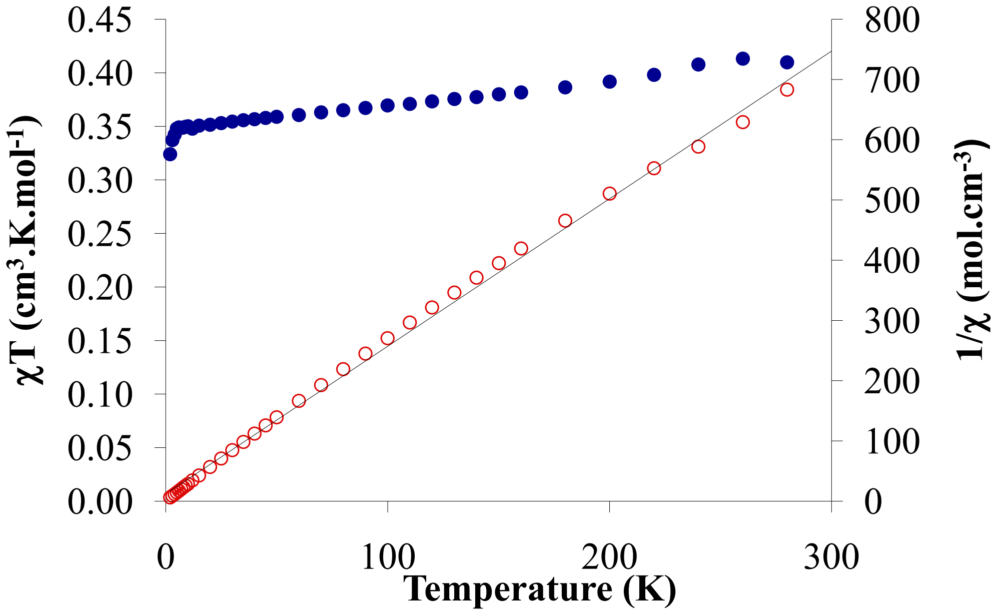

Figure S16. Temperature dependant magnetic data for **2.** Plot of χT vs. T (blue dots) and 1/χ vs. T (red unfilled dots).

C.3. Electron Paramagnetic Resonance (EPR) Spectroscopy

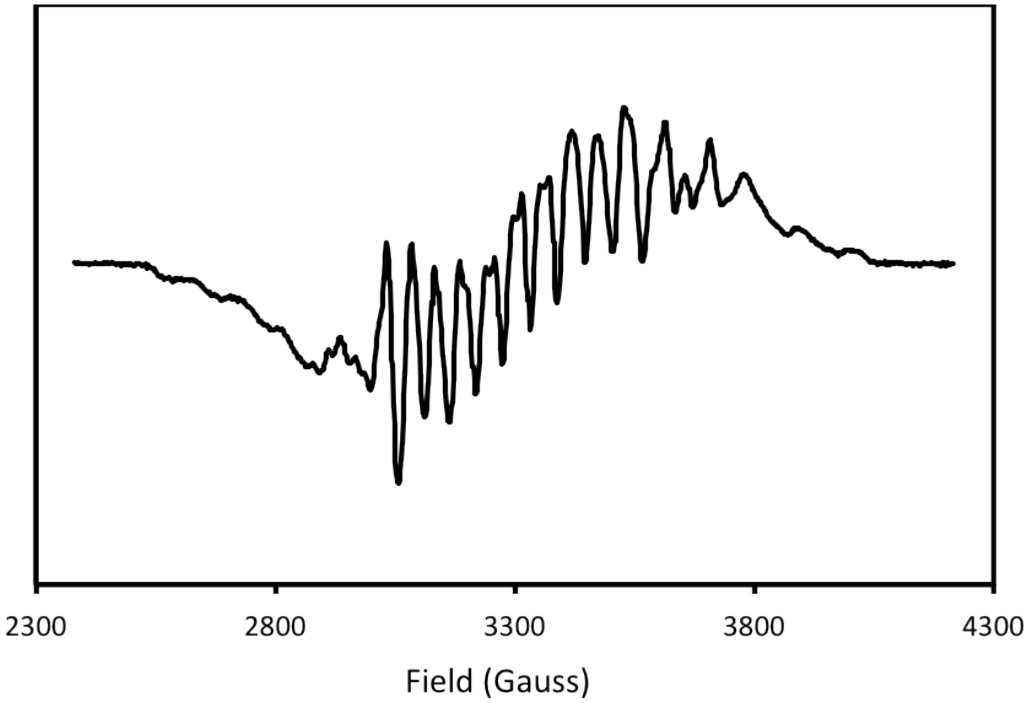

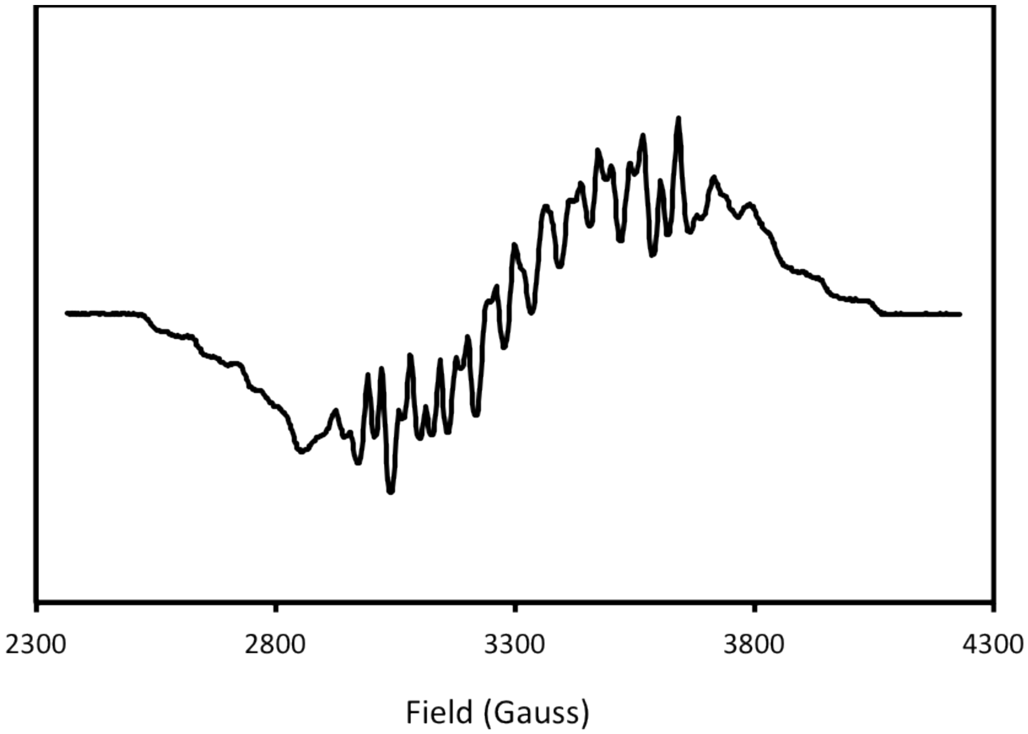

Figure S17. Experimental X-Band EPR of **2** recorded in solid state at 300K (top) and 4K (bottom).

D. DFT calculation

D.1. TD-DFT calculation

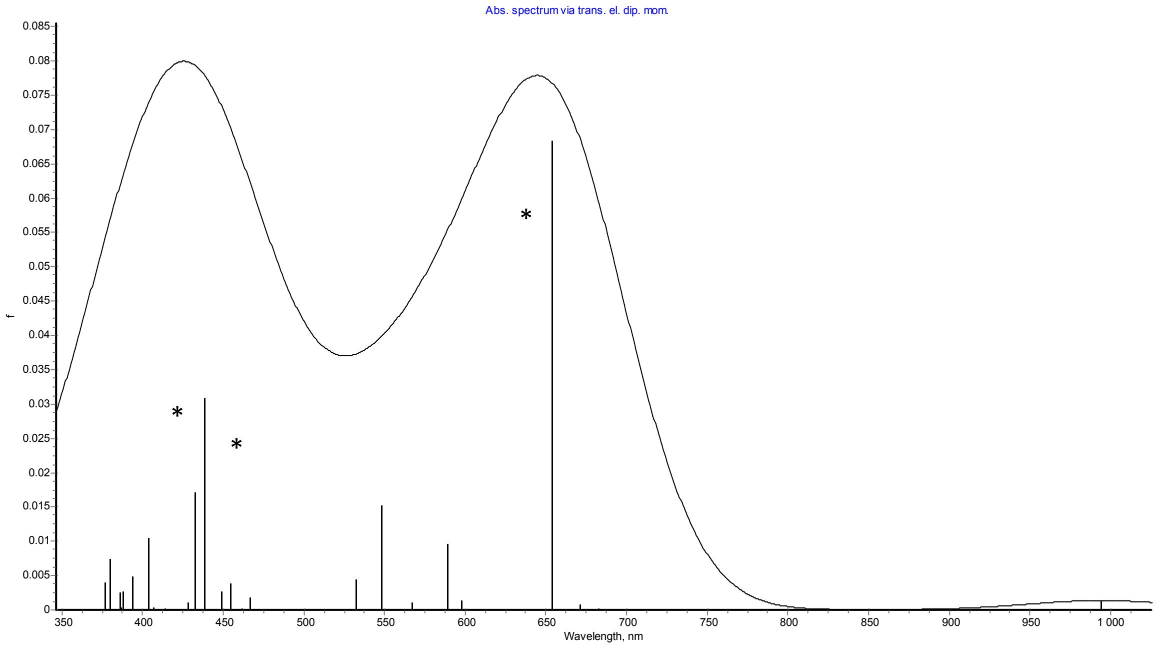

Figure S18. Calculated transition from TD-DFT for **2-I**. The line was obtained using a substantial Doppler broadening and * correspond to the three main transitions discussed in the text.

D.2. DFT optimized Cartesian atomic coordinates of 1 and 2.

Complex1

C	0.989961	0.356613	0.040356
C	0.553186	0.413083	-1.294681
H	-0.196908	1.180579	-1.492151
C	1.059629	-0.215990	-2.440854
C	0.382864	1.413943	0.940825
H	-0.632115	1.084078	1.220994
H	0.279841	2.371233	0.410777
H	0.957288	1.572036	1.862255
C	0.517718	0.314669	-3.751929
H	1.197070	0.138504	-4.595690
H	0.298564	1.388889	-3.681894
H	-0.430755	-0.204713	-3.970455
C	2.425047	-0.315170	1.815699
C	3.621311	0.440698	1.895846
C	4.218809	0.639466	3.143820
H	5.140428	1.218493	3.208583
C	3.665074	0.116061	4.304983
H	4.144616	0.283779	5.270730
C	2.491601	-0.620212	4.219053
H	2.046998	-1.024036	5.130366
C	1.858014	-0.850472	2.994398
C	0.559382	-1.634877	2.997219
H	0.283535	-1.807900	1.948196
C	4.252197	1.074019	0.667904
H	3.955407	0.461447	-0.195962
C	2.563732	-1.603816	-3.662178
C	2.021154	-2.599939	-4.503462
C	2.681361	-2.915486	-5.694818
H	2.254681	-3.676932	-6.350497
C	3.854111	-2.275452	-6.067937
H	4.353223	-2.533598	-7.003291
C	4.385394	-1.300417	-5.234047
H	5.311961	-0.803519	-5.520623
C	3.763291	-0.948855	-4.033544
C	4.376037	0.143073	-3.174068
H	4.098237	-0.081044	-2.133614
C	0.724282	-3.321579	-4.194653
H	0.382126	-2.974424	-3.211328
C	-1.047078	-3.437648	-0.364433
C	-1.098302	-4.519509	0.725754
H	-0.506338	-5.397337	0.424963
H	-2.139156	-4.839891	0.900616
H	-0.684968	-4.134518	1.665715
C	-1.621524	-4.023131	-1.663325
H	-1.647391	-3.261907	-2.455909
H	-2.650678	-4.379916	-1.491908
H	-1.009187	-4.870471	-2.000639
C	-1.899996	-2.227077	0.050474
H	-1.566985	-1.825410	1.017376

H	-2.958442	-2.523383	0.143248
H	-1.814680	-1.429011	-0.702671
C	8.863195	-6.825656	-1.541042
H	8.936058	-6.334559	-2.519563
H	9.043699	-7.902713	-1.663069
H	9.667585	-6.427705	-0.899089
C	7.539739	-6.571127	-0.848971
C	7.208618	-7.504758	0.144071
H	7.966207	-8.270719	0.317098
C	5.989623	-7.728937	0.806021
C	5.928077	-9.022101	1.593797
H	6.392173	-8.851167	2.580021
H	6.499912	-9.815858	1.093503
H	4.900291	-9.368416	1.761791
C	3.686667	-7.358457	1.280819
C	2.781864	-7.957255	0.368940
C	1.521385	-8.354770	0.824327
H	0.819843	-8.811469	0.125797
C	1.139802	-8.180417	2.148762
H	0.149684	-8.493306	2.484176
C	2.038739	-7.611415	3.039769
H	1.751206	-7.488747	4.085716
C	3.311063	-7.198997	2.633052
C	4.249264	-6.631659	3.680258
H	5.172339	-6.335533	3.165225
C	3.163929	-8.217146	-1.077983
H	3.886751	-7.438199	-1.361836
C	7.101403	-4.803951	-2.384759
C	6.526358	-5.263361	-3.595348
C	6.829484	-4.601835	-4.787955
H	6.384346	-4.951211	-5.719463
C	7.681090	-3.506003	-4.812805
H	7.911787	-3.006097	-5.754990
C	8.232678	-3.055565	-3.622348
H	8.909203	-2.199111	-3.637566
C	7.958013	-3.680299	-2.401904
C	8.643142	-3.141067	-1.160132
H	8.238667	-3.690638	-0.299748
C	5.611779	-6.474719	-3.639161
H	5.137711	-6.550020	-2.649832
C	7.573165	-4.031241	2.637623
C	7.784790	-2.509018	2.673770
H	6.844065	-2.001323	2.936945
H	8.115216	-2.145075	1.693425
H	8.547884	-2.245630	3.425311
C	7.080797	-4.483637	4.020090
H	6.960894	-5.575763	4.051539
H	6.117191	-4.009814	4.250018
H	7.810365	-4.192539	4.794052
C	8.897815	-4.746580	2.322836
H	8.728840	-5.831271	2.241352
H	9.631307	-4.555840	3.124269
H	9.322049	-4.391106	1.373761
C	2.932170	-4.665004	-0.652830
H	2.331541	-5.516759	-0.965980
C	3.701368	-3.931678	-1.633641

H	3.792023	-4.304754	-2.654071
C	4.685126	-2.958132	-1.142539
H	5.249447	-2.367881	-1.864836
C	4.552413	-2.510286	0.225800
H	5.139695	-1.663678	0.576713
C	3.561043	-3.081061	1.135999
H	3.590103	-2.772851	2.182110
C	3.053675	-4.402554	0.780984
H	2.375365	-4.924922	1.457489
N	1.841044	-0.552834	0.525397
N	1.929211	-1.234842	-2.424360
N	0.323878	-3.027630	-0.553393
N	4.952486	-6.885790	0.794868
N	6.793866	-5.504763	-1.165455
N	6.595765	-4.346231	1.624358
Nb	2.069661	-2.568570	-0.606533
Nb	5.350003	-4.639306	0.350247
C	10.162384	-3.362592	-1.221468
H	10.608894	-2.776425	-2.041808
H	10.636047	-3.037417	-0.280777
H	10.415948	-4.418293	-1.392296
C	8.350156	-1.650477	-0.950685
H	8.883183	-1.276017	-0.062451
H	8.688303	-1.049337	-1.809825
H	7.275691	-1.469553	-0.811133
C	4.594508	-7.684022	4.745845
H	5.369726	-7.295203	5.426089
H	3.708561	-7.929876	5.354336
H	4.958713	-8.619365	4.297327
C	3.666782	-5.386325	4.358616
H	2.678411	-5.595375	4.798490
H	4.328856	-5.049848	5.171487
H	3.556898	-4.559651	3.644917
C	6.394191	-7.775117	-3.880373
H	7.085356	-7.998610	-3.057389
H	5.697556	-8.623789	-3.976192
H	6.976904	-7.701131	-4.814217
C	4.509347	-6.347073	-4.696470
H	3.753677	-7.132811	-4.545261
H	4.009374	-5.368229	-4.659137
H	4.913371	-6.481437	-5.713398
C	3.862582	-9.576773	-1.239721
H	4.074890	-9.769273	-2.303803
H	4.814494	-9.615250	-0.694381
H	3.213223	-10.385979	-0.864963
C	1.973848	-8.149690	-2.041210
H	1.309524	-9.020838	-1.921186
H	1.368397	-7.241775	-1.901182
H	2.334804	-8.162042	-3.080456
C	3.720342	2.496721	0.432117
H	4.230576	2.954398	-0.430695
H	2.641971	2.501940	0.227933
H	3.908757	3.125504	1.318755
C	5.904881	0.185110	-3.268005
H	6.310789	0.830463	-2.474242
H	6.354310	-0.813848	-3.170299

H	6.234089	0.612344	-4.229584
C	3.811705	1.530787	-3.516905
H	4.335553	2.305792	-2.933979
H	3.959782	1.748069	-4.588326
H	2.740225	1.608107	-3.290626
C	5.783523	1.116996	0.730545
H	6.222208	0.149441	1.016574
H	6.188364	1.400734	-0.252543
H	6.135346	1.871877	1.452377
C	0.709434	-2.998993	3.680427
H	1.045848	-2.892619	4.724056
H	-0.258927	-3.523470	3.701013
H	1.431779	-3.636085	3.154322
C	-0.565101	-0.841343	3.681241
H	-1.521413	-1.382913	3.597101
H	-0.347433	-0.707774	4.754003
H	-0.690213	0.157374	3.240693
C	0.927272	-4.839396	-4.114080
H	-0.031434	-5.343640	-3.916191
H	1.322278	-5.240858	-5.061357
H	1.629763	-5.103766	-3.312491
C	-0.358580	-3.000962	-5.236002
H	-0.087572	-3.411286	-6.222837
H	-1.318907	-3.452760	-4.938688
H	-0.503593	-1.917818	-5.356626

Complex2 :

C	11.945953	4.640686	10.692994
H	11.527975	4.437958	9.699365
H	11.526587	5.595244	11.053912
H	11.616210	3.857059	11.389019
C	13.452737	4.758875	10.686387
C	14.065383	4.584463	11.938702
H	13.363458	4.428205	12.760570
C	15.408561	4.344823	12.280570
C	15.616070	3.779161	13.666140
H	15.077396	2.825247	13.771963
H	15.181945	4.474246	14.403110
H	16.672772	3.616761	13.908287
C	17.726607	4.006282	11.791743
C	17.959166	2.628165	11.575542
C	19.223023	2.106860	11.869937
H	19.407483	1.043160	11.711536
C	20.239871	2.905928	12.369646
H	21.216346	2.477400	12.600302
C	19.993897	4.253490	12.598032
H	20.785461	4.877844	13.013587
C	18.748533	4.824863	12.328336
C	18.514883	6.273161	12.714593
H	17.624164	6.623479	12.175817
C	18.226715	6.382625	14.220814
H	17.330485	5.815895	14.509588
H	18.077192	7.435394	14.508035
H	19.076740	5.987894	14.801630

C	19.683432	7.195790	12.351397
H	20.559715	7.010255	12.992809
H	19.392410	8.246679	12.508355
H	20.006374	7.072274	11.307743
C	16.888189	1.663268	11.094164
H	15.965657	2.232596	10.910794
C	17.286855	0.974211	9.783536
H	17.404099	1.693998	8.960987
H	16.517837	0.241608	9.493343
H	18.236832	0.427250	9.894346
C	16.583577	0.599871	12.162068
H	17.442803	-0.076325	12.298028
H	15.721022	-0.012111	11.854557
H	16.360477	1.051032	13.139020
C	13.412277	4.887374	8.299970
C	12.753503	5.985835	7.703788
C	12.035155	5.775358	6.522523
H	11.509837	6.615434	6.065729
C	11.953470	4.522105	5.934729
H	11.366702	4.375147	5.026637
C	12.628319	3.455840	6.513681
H	12.576657	2.475552	6.040367
C	13.373752	3.612854	7.685119
C	14.089266	2.407628	8.273139
H	14.949244	2.788109	8.846706
C	13.192313	1.624188	9.244005
H	12.906786	2.224928	10.117812
H	13.719122	0.726848	9.607028
H	12.270612	1.296798	8.734755
C	14.618932	1.458478	7.192033
H	13.800833	0.909029	6.699995
H	15.281281	0.707684	7.645611
H	15.182782	1.995035	6.414284
C	12.735892	7.379124	8.305730
H	13.412624	7.374717	9.170416
C	13.232854	8.440779	7.315195
H	14.239956	8.219194	6.934985
H	13.249719	9.430734	7.798201
H	12.562753	8.516573	6.444177
C	11.327422	7.757691	8.790528
H	10.626616	7.812239	7.941719
H	11.340023	8.747335	9.274644
H	10.925500	7.026377	9.504956
C	15.167892	8.639707	11.413239
C	16.043428	8.838474	12.657086
H	15.926770	7.993267	13.349750
H	15.743850	9.761303	13.179133
H	17.102108	8.927632	12.375288
C	13.698272	8.521157	11.844060
H	13.037631	8.425163	10.972564
H	13.401178	9.422254	12.404539
H	13.560799	7.640970	12.489696
C	15.349909	9.828406	10.458096
H	16.406938	9.932233	10.168832
H	15.033305	10.761483	10.951607
H	14.746105	9.690378	9.550377

C	22.941970	5.524167	5.308548
H	23.463486	5.783772	6.238487
H	23.323037	6.145810	4.486312
H	23.171761	4.472434	5.068072
C	21.450519	5.675989	5.449854
C	20.725233	5.727708	4.223031
H	21.355872	5.460186	3.369924
C	19.460070	6.229085	3.807732
C	19.317840	6.491959	2.331515
H	19.152698	5.532173	1.813794
H	20.232621	6.940228	1.919784
H	18.460972	7.144370	2.120689
C	17.240240	7.085395	4.249540
C	16.176213	6.399939	3.628585
C	15.013126	7.117010	3.328346
H	14.182358	6.600498	2.845646
C	14.896250	8.467030	3.623506
H	13.981029	9.007303	3.377150
C	15.958298	9.130265	4.227428
H	15.863092	10.192553	4.451710
C	17.141567	8.463428	4.550619
C	18.304391	9.226334	5.159005
H	18.845174	8.523390	5.812468
C	19.278628	9.701240	4.068745
H	19.731255	8.861289	3.524232
H	20.094202	10.291900	4.514632
H	18.751483	10.337491	3.338642
C	17.872598	10.424800	6.008061
H	17.472269	11.239707	5.384616
H	18.743325	10.832489	6.543253
H	17.107067	10.154800	6.750292
C	16.233593	4.930630	3.250378
H	17.192852	4.518828	3.596509
C	16.158726	4.738066	1.727866
H	15.173540	5.047622	1.343906
H	16.297661	3.675265	1.473638
H	16.919319	5.327108	1.195935
C	15.114533	4.132410	3.928964
H	15.146120	4.233944	5.022415
H	15.197408	3.064676	3.671513
H	14.123310	4.476982	3.594613
C	21.629625	5.785594	7.854771
C	21.791189	7.061098	8.442866
C	22.502526	7.153713	9.640747
H	22.643383	8.128927	10.106252
C	23.034316	6.026173	10.255810
H	23.588587	6.121507	11.190738
C	22.865457	4.783072	9.665443
H	23.292453	3.900026	10.143052
C	22.174318	4.633987	8.458659
C	22.074313	3.246596	7.851614
H	21.508160	3.322579	6.913288
C	23.462645	2.673482	7.527762
H	24.062172	3.358470	6.911285
H	23.364085	1.716516	6.991059
H	24.030332	2.477901	8.451366

C	21.315388	2.281353	8.770063
H	21.855345	2.136726	9.719308
H	21.218011	1.295337	8.288206
H	20.311880	2.656995	9.013072
C	21.252048	8.314839	7.778353
H	20.312251	8.032679	7.277241
C	22.220652	8.835931	6.704875
H	23.210091	9.035198	7.148817
H	21.842478	9.776257	6.273830
H	22.352194	8.119191	5.882518
C	20.939642	9.440655	8.768344
H	20.347056	9.088549	9.625591
H	20.374967	10.236597	8.260026
H	21.860589	9.901130	9.160122
C	19.058033	1.777772	5.030608
C	20.535283	1.476036	4.739248
H	20.946072	2.218519	4.038330
H	20.639070	0.475194	4.289646
H	21.123162	1.501215	5.666993
C	18.512353	0.770546	6.053909
H	19.068083	0.845903	6.999628
H	18.612183	-0.256238	5.665789
H	17.449349	0.964820	6.254728
C	18.246800	1.691481	3.730601
H	17.191526	1.928038	3.926031
H	18.305264	0.676285	3.305813
H	18.637756	2.402534	2.987173
C	16.987225	4.852522	7.240640
C	18.151206	4.640755	8.190664
C	18.520064	5.764562	9.035642
C	18.021989	7.048056	8.801305
C	16.907360	7.242947	7.910066
C	16.326102	6.147447	7.270044
N	16.442757	4.570172	11.457038
N	14.121889	5.025971	9.550075
N	15.569625	7.429817	10.727593
N	20.856878	5.684824	6.646481
N	18.431141	6.387995	4.648250
N	18.941129	3.108025	5.587968
Nb	18.913881	4.823934	6.101694
Nb	16.092186	6.002809	9.794275
H	18.280574	3.648298	8.630276
H	16.352254	3.988971	7.023837
H	18.413481	7.901184	9.356062
H	15.431303	6.290213	6.659513
H	16.474914	8.237705	7.804440
H	19.305335	5.612393	9.779070
